# Interferon-Regulated Expression of Cellular Splicing Factors Modulates Multiple Levels of HIV-1 Gene Expression and Replication

**DOI:** 10.3390/v16060938

**Published:** 2024-06-11

**Authors:** Fabian Roesmann, Lisa Müller, Katleen Klaassen, Stefanie Heß, Marek Widera

**Affiliations:** 1 Institute for Medical Virology, University Hospital Frankfurt, Goethe University Frankfurt, Paul-Ehrlich-Str. 40, 60596 Frankfurt am Main, Germany; 2 Institute of Virology, Medical Faculty, University Hospital Düsseldorf, Heinrich-Heine-University Düsseldorf, Universitätsstr. 1, 40225 Düsseldorf, Germany

**Keywords:** HIV-1, SRSF, hnRNP, viral replication, RNA splicing, RNA processing, programmed ribosomal frameshifting (-1PRF), interferon-repressed genes (IRepGs), interferon-stimulated genes (ISGs), antiviral state, PBMCs, LPMCs

## Abstract

Type I interferons (IFN-Is) are pivotal in innate immunity against human immunodeficiency virus I (HIV-1) by eliciting the expression of IFN-stimulated genes (ISGs), which encompass potent host restriction factors. While ISGs restrict the viral replication within the host cell by targeting various stages of the viral life cycle, the lesser-known IFN-repressed genes (IRepGs), including RNA-binding proteins (RBPs), affect the viral replication by altering the expression of the host dependency factors that are essential for efficient HIV-1 gene expression. Both the host restriction and dependency factors determine the viral replication efficiency; however, the understanding of the IRepGs implicated in HIV-1 infection remains greatly limited at present. This review provides a comprehensive overview of the current understanding regarding the impact of the RNA-binding protein families, specifically the two families of splicing-associated proteins SRSF and hnRNP, on HIV-1 gene expression and viral replication. Since the recent findings show specifically that SRSF1 and hnRNP A0 are regulated by IFN-I in various cell lines and primary cells, including intestinal lamina propria mononuclear cells (LPMCs) and peripheral blood mononuclear cells (PBMCs), we particularly discuss their role in the context of the innate immunity affecting HIV-1 replication.

## 1. The High Complexity of the HIV-1 Genome Requires Exceptional Expression Strategies

**Figure 1:**
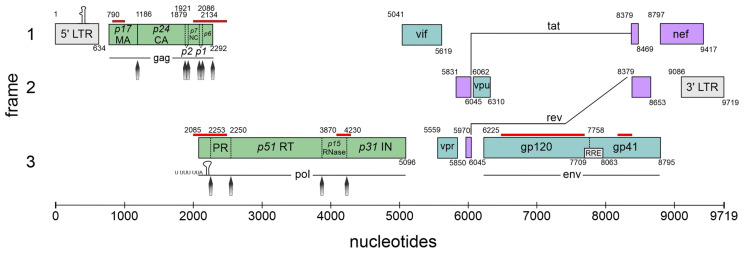
The complexity of the HIV-1 genome. Schematic overview of the nine major open reading frames (ORFs) encoded by HIV-1 using all three open reading frames (frames 1–3). The long terminal repeats (LTRs) are located at the terminal ends of the integrated genome serving as provirus. The trans-activation response (TAR) element is indicated at the 5′ LTR (gray box). The stem-loop and respective slippery sequence (U UUU UUA), required for programmed ribosomal frameshifting, is indicated at the frameshift site of the *pol* mRNA. The Rev-response element (RRE) within the *env*-coding region is indicated as a small box. *Cis*-acting repressive sequences (CRSs) according to [3] are shown in red lines above the respective sequence. Protease cleavage sites are indicated with gray arrows. The gene products encoded by the respective HIV-1 mRNA classes are represented in the following colors: 9 kb in green, 4 kb in turquoise, 2 kb in purple. Nucleotide positions are referenced relative to HXB2 (GenBank accession number K03455). Illustration and protease cleavage sites are adapted from ViralZone/SwissBioPics [4].

In contrast to host cells, which predominantly comprise non-coding and regulatory sequences, viruses exhibit a constrained genomic capacity. The loci of the host genome are spatially separated and use a rather linear pathway for gene expression [1] implicating that one locus is transcribed and then translated into a single protein with multiple isoforms. Viruses such as human immunodeficiency virus I (HIV-1), with their much smaller but complex genomes (Figure 1), use unconventional pathways to use the full potential of their limited genome size. This involves regulation on the level of transcription, RNA processing and splicing, trafficking, as well as translation to generate several protein products from a single locus. In this context, viruses rely on utilizing the cellular machinery, for which they depend on the associated host cell proteins [2]. Since these cellular host factors are necessary for replication, they are referred to as dependency factors.

**Figure 2:**
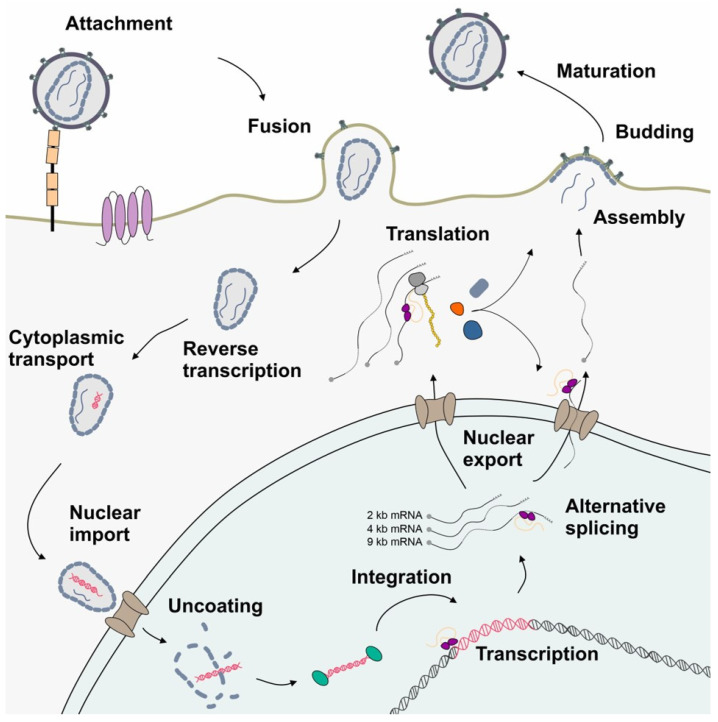
A schematic drawing depicting the simplified HIV-1 replication cycle highlighting post-integration steps. Following attachment and glycoprotein-mediated membrane fusion, the viral particle is transported to the nuclear pore complex, while genomic RNA is reverse-transcribed into dsDNA. The viral dsDNA is integrated into the host genome serving as provirus. LTR-driven transcription by the RNAPII enables the synthesis of the HIV-1 full-length pre-mRNA, which subsequently undergoes extensive alternative splicing. This process maintains an equilibrium of protein coding mRNA isoforms of 9, 4, and 2 kb size, which are then exported into the cytoplasm and serve as a template for the translation of viral proteins. The newly assembled virions exit the host cell by budding. The extracellular space is shown in white, the cytoplasm in gray, and the nucleus in aquamarine.

HIV-1 is the causative agent of acquired immunodeficiency syndrome (AIDS) and belongs to the *Retroviridae* family (genus lentivirus) categorized in Baltimore classification group VI. This group comprises positive-sense single-stranded (ss) RNA viruses that use reverse transcription during their replication cycle (Figure 2) for the synthesis of the positive double-stranded (ds) DNA, whose integration as a provirus into the host cell genome is catalyzed by the viral enzyme integrase (reviewed in [5,6,7]). This stable and irreversible integration is essential for establishing a persistent viral reservoir within the host, enabling long-term viral replication and persistence. Once integrated, the viral DNA becomes a permanent part of the host cell genome, and the viral gene expression can be regulated alongside the host cellular genes. As discussed below (Section 4), the long terminal repeats (LTRs) flank the integrated viral genome and serve as promoters for the viral sense (and antisense [8]) transcription mediated by RNA polymerase II (RNAPII). This leads to the generation of viral pre-mRNAs, which subsequently undergo splicing, capping, and polyadenylation. Initially, the transcription efficiency is minimal and necessitates enhancement through trans-activation by the HIV-1-encoded trans-activator of transcription (Tat) [5,9,10]. The primary full-length pre-mRNA undergoes excessive alternative splicing (Section 5), leading to a spatiotemporal equilibrium between the spliced and unspliced mRNAs, crucial for efficient viral replication [11,12,13]. The unspliced 9 kb mRNA functions as the genomic RNA and is encapsulated into newly formed virions. The 9 kb mRNA also encodes for the first ORF of the group-specific antigen (Gag) that harbors the structural proteins essential for viral assembly, including matrix (MA, p17), capsid (CA, p24), nucleocapsid (NC, p7), and p6. Also, the second ORF polymerase (Pol), whose expression depends on a frameshifting event, is encoded by the full-length 9 kb mRNA and harbors the viral enzymes’ reverse transcriptase (RT, p51), integrase (IN, p31), and protease (PR). The regulatory protein Tat encoded by the 2 kb mRNA class-derived *tat* gene is decisively involved in the regulation of viral transcription. Tat changes the chromatin conformation at the proviral integration site, making it more available for transcription by binding to the TAR elements at the 5′ end of the viral leader mRNA [14]. Tat facilitates the recruitment of P-TEFb, leading to the hyperphosphorylation of RNAPII, which significantly increases the processivity of the enzymatic complex [15,16,17]. In addition, Tat trans-activates the transcription of viral RNAs via NF-κB activating by modulating the cellular redox state and IκB-α degradation [18,19,20].

The 2 kb mRNA also encodes the regulator of expression of virion proteins (Rev), which is an RNA-binding phosphoprotein expressed during the early stages of HIV-1 replication [21,22]. Rev facilitates the nuclear export of unspliced 9 kb and intron-containing 4 kb viral mRNA isoforms, which is crucial for viral gene expression [23,24]. The arginine-rich domain of Rev mediates RNA binding and nuclear localization [25]. Homo-multimerized Rev interacts with the nucleolar phosphoprotein B23 and importin β via its NLS domain [26]. This interaction enables a highly complex cycle of protein shuttling where even small amounts of Rev are capable of mediating the export of the intron-containing viral mRNAs. Aside from nuclear export facilitation, Rev increases the stability and translation of viral RNAs [27,28]. The negative factor (Nef) modulates various cellular processes to promote viral replication and immune evasion, including the downregulation of CD4, CD8, CD28, and major histocompatibility complex (MHC) class I and II molecules and upregulation of CD74 [29,30,31,32]. Moreover, Nef disrupts the signal transduction pathways by forming complexes with PAK-2 and interacting with lipid rafts, which serve as hubs for signaling mediators [33,34]. Nef also induces actin remodeling and facilitates the translocation of the viral core across the obstructive actin barrier, resulting in increased virion infectivity and replication [35]. In addition, the cholesterol trafficking in infected cells is regulated by Nef binding, which influences the HIV assembly, budding, and infection of target cells. Hence, the downregulation of cellular cholesterol reduces HIV-1 particle production [36].

The 4 kb mRNA encodes the accessory proteins Vif, Vpr, Vpu, and envelope (Env). The viral infectivity factor (Vif) is crucial for viral replication since it counteracts APOBEC3G-mediated host restriction, maintaining the infectivity of newly assembled virions by preventing the subsequent hypermutation of the viral genome during reverse transcription [37,38,39]. The pleiotropic viral protein R (Vpr) is involved in multiple steps of viral replication, including facilitating the nuclear import of the viral pre-integration complex, inducing cell cycle arrest, and modulating the host immune responses [40,41]. Viral protein U (Vpu) enhances the viral release by antagonizing tetherin/BST-2, which inhibits the release of nascent virions from infected cells. Further, Vpu induces the degradation of the CD4 receptor by ubiquitination via the recruitment of SKP1 [42]. Env codes for the viral envelope glycoprotein precursor gp160, including surface glycoprotein gp120 and transmembrane glycoprotein gp41, crucial for the viral entry and fusion with the host cells. Moreover, gp120 binds to the CD4 receptor, triggering the conformational changes of the gp120/gp41 trimer and exposing the CCR5 and CxCR4 co-receptor binding site of gp120. The binding of the co-receptors enables glycoprotein-mediated membrane fusion and cellular entry. As the sole surface protein of HIV-1, Env represents the primary target for the host adaptive immune system, prompting antibody production; its expression is tightly regulated to balance efficient viral entry with the evasion of the host immune responses [43].

HIV-1 represents a perfect model for the study of host dependency factors as it includes multiple layers of unconventional gene expression, including reverse transcription and integration into the host genome followed by the LTR transcription of full-length mRNA, alternative splicing, and the export of intron-containing mRNAs. The use of all three open reading frames (Figure 1) requires programmed ribosomal frameshifting (-1PRF) and leaky scanning. Furthermore, polyprotein synthesis requires post-translational processing. In this review, we will focus on the post-integration steps up to translational initiation and -1PRF and describe how interferons might affect HIV-1 gene expression.

## 2. HIV-1 Depends on Interactions with Host Cell Splicing Factors

Following the integration of proviral DNA into the host chromosome, the 5′LTR functions as the viral promoter, initiating the synthesis of viral mRNA. Eukaryotic translation, however, adheres to the principle of ribosomal scanning, where the ribosomes enter the 5′ end of a capped mRNA and traverse off the strand until the first AUG start codon is encountered, which initiates the synthesis of the Gag polyprotein. A major level of the HIV-1 gene expression strategy involves positioning each of its nine open reading frames proximal to the 5′ end, which is accomplished through alternative splicing (Section 5). HIV-1 produces a plethora of mRNA transcripts via the alternative utilization of multiple splice donor and acceptor sites, resulting in the generation of more than 50 mRNA isoforms yielding at least 15 viral protein isoforms [11,44,45] (also reviewed in [43]). By binding to the RRE (Figure 1) and recruiting additional host cellular factors (see Section 6), Rev facilitates the nucleocytoplasmic export of full-length 9 kb mRNA and isoforms of the intron-containing 4 kb class.

The two types of RNA-binding protein (RBP) families named heterogeneous nuclear ribonucleoproteins (hnRNPs) and serine/arginine-rich splicing factors (SRSFs) play crucial roles in regulating HIV-1 gene expression. They regulate alternative splicing events, enabling the production of all the necessary viral proteins in balanced ratios, essential for efficient HIV-1 replication and infectivity [43]. In particular, SRSFs and hnRNPs modulate the alternative splice site use by binding to sequence-specific *cis*-regulatory RNA sequences, facilitating or impeding the use of a specific splice site in a sequence- and position-dependent manner, thereby decisively regulating the mRNA isoform levels. Recent studies, however, demonstrate that members of the two protein families not only determine the alternative splice site use, as their names imply, but also exert regulatory control over numerous RNA processing events beyond splicing, including mRNA stability, localization, and translation efficiency, in sum contributing to the fine-tuning of HIV-1 gene expression.

In the following sections, we describe the characteristics and functional hallmarks of these two protein families and discuss their impact on viral replication but focus on two representatives, which have recently been shown to be significantly modulated by interferons [46,47]. SRSF1 will be examined as the main representative of the SRSF family, and hnRNPA0 will be discussed as a representative of the hnRNP family, both selected due to their significant downregulation in gene expression upon interferon stimulation among all the examined RBPs (Table 1).

### 2.1. Serine/Arginine-Rich Splicing Factors

**Figure 3:**
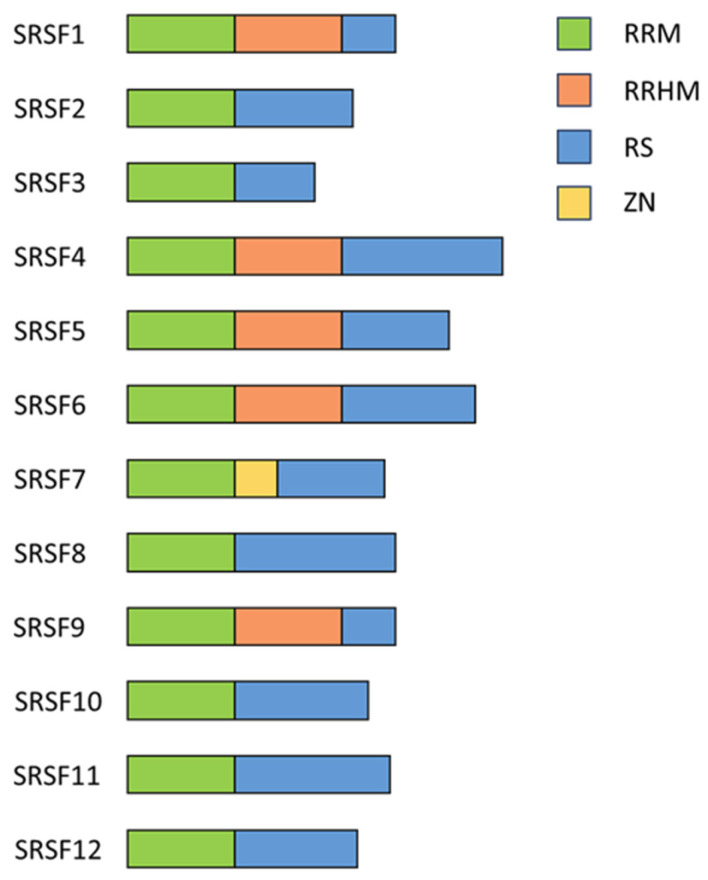
Schematic drawing of the structural overview of SRSF proteins. A schematic representation illustrating the modular organization and domain architecture of SRSF proteins. SRSF proteins typically consist of one or more RRMs responsible for RNA binding, followed by a serine/arginine-rich (RS) domain crucial for protein–protein interactions and splicing regulation. Auxiliary domains, RS-like domain, ZnF domain, or RRM-like motifs are depicted in the indicated colors. Illustration adapted from [69].

In the early 1990s, a large family of RNA-binding proteins (RBPs) containing serine- and arginine-rich dipeptide repeat (RS-) domains (Figure 3) was first described [48], and its members have since been described as multifaceted regulators of gene expression [49]. Due to the presence of the RS domain, they have been uniformly renamed to serine/arginine-rich splicing factors 1–12 (SRSFs) [50]. They harbor a highly conserved RNA recognition motif (RRM), necessary for interacting with the pre-mRNAs and RS domains, functioning as protein–protein-interactors [51]. Most SRSF proteins are ubiquitously expressed with low tissue specificity; however, SRSF5 and SRSF12 are exceptions, being enriched in monocytes and testes, respectively [52].

SRSF proteins are essential for multiple steps of cellular and viral gene expression, including pre-mRNA splicing, alternative splice site selection, intron removal, and exon ligation through binding to the *cis*-regulatory elements in the HIV-1 genome [43,53] (see Section 5). Additionally, they participate in regulating other RNA-mediated processes, such as RNA stability, export, and translation.

Post-translational modifications (PTMs) like phosphorylation [54], methylation [55,56,57], and acetylation [58] are decisive for SRSF protein activity and subcellular localization. The roles of SR proteins include the interaction with interphase chromatin via SRSF1 and SRSF3 [59], transcriptional regulation by SRSF2 [60], nuclear mRNA export adaptation by SRSF3 and SRSF7 [61,62], the regulation of mRNA decay [63], and the maintenance of genome stability [64]. The phosphorylation of the serine residues within the RS domain primarily regulates its protein–RNA and protein–protein interactions, as well as protein stability [65,66]. Depending on the phosphorylation state, SRSFs are localized within the nucleus, nuclear speckles, or cytoplasm [67,68]. The main kinases phosphorylating the SRSF protein family are the SR protein-specific kinases (SRPKs), Cdc2-like kinases (CLKs), and dual-specificity tyrosine phosphorylation-regulated kinases (DYRKs) [69,70]. Those kinases have different subcellular localizations, with CLKs primarily associating with nuclear speckles. Upon phosphorylation by CLKs, SRSF proteins are released from the nuclear speckles into the nucleoplasm [69]. SRPKs, primarily cytosolic proteins, phosphorylate SRSFs shortly after synthesis, regulating their re-localization to nuclear speckles [69]. Consequently, these kinases influence the SR protein activity in RNA metabolism and alternative splicing [71,72]. Altering the levels of SRSF phosphorylating proteins CLK1 and CLK2 influences the HIV-1 gene expression and even suppresses the replication of other viruses like coronaviruses [73]. Hence, targeting phosphorylation represents a promising target for antiviral therapy [74] (Section 8).

Dysfunctions in SRSF proteins are implicated in various human diseases, including cancer, neurological disorders, and cardiovascular diseases [49]. Therefore, understanding the functions and regulation of SRSF expression is crucial for elucidating the cellular and viral RNA processing and their roles in the disease pathogenesis.

### 2.2. Serine/Arginine-Rich Splicing Factor 1

The protein SRSF1, formerly known as SRp30 or ASF/SF2 [50], has been one of the founding members of the SRSF protein family. It was identified in the early 1990s as an enhancer of the spliceosomal assembly and a regulator of the pre-mRNA splicing in HeLa cells and alternative splicing of the SV40 pre-mRNA in HEK293 cells [75,76]. SRSF1 comprises an RRM, an RRM homology domain (RRMH), and an RS domain (Figure 3) [69] and is a pleiotropic protein involved in transcription activation [77], alternative splicing [43,69,78,79], mRNA stability and export [80], and mRNA translation [81,82]. It can also induce nonsense-mediated mRNA decay (NMD), a mechanism that degrades newly synthesized mRNAs containing premature termination codons [83,84,85]. SRSF1 is also involved in maintaining genome stability [64]. Using the selected evolution of ligands through the exponential enrichment (SELEX) method, purine-rich sequences were identified as high-affinity binding sites for SRSF1 [86]. In agreement, the CLIP-seq analysis (cross-linking immunoprecipitation and high-throughput sequencing) of the HEK293T transcriptome revealed 23,632 binding sites for SRSF1 with a consensus sequence of GGAGA within the exonic regions [87,88], and many were in close proximity to splice sites [87], highlighting its role in alternative splice site use.

SRSF1 was also described as a proto-oncogene since it is involved in the splicing of many cancer-related genes [89,90,91]. SRSF1 knockout has been shown to be lethal in mouse embryos, while its overexpression causes oncogenesis in both human and rodent cells [92]. SRSF1 is also a key regulator in the gene expression and RNA processing of HIV-1 [78,79,93]. The overexpression of SRSF1 has been shown to suppress viral transcription, while the knockdown increases the total viral mRNA levels [46,94,95,96]. As mentioned above, several HIV-1 pre-mRNA SREs are bound by SRSF1, which enhances the recruitment of the core spliceosomal components [92].

Undergoing various PTMs, SRSF1 experiences regulatory changes affecting both its subcellular localization and functional repertoire (reviewed in [92]). Here, the transitions between the phosphorylation states modulate the interactions of SRSF1 with other proteins, its RNA-binding characteristics and target specificity, as well as subcellular localization. These modifications encompass extensive phosphorylation events targeting the Ser residues within the RS domain [67,68,97]. Enzymes such as Clk/Sty kinases 1–4 execute the phosphorylation within the nucleus, while SRPK 1–2 predominantly mediate cytoplasmic phosphorylation. Additionally, topoisomerase 1 and phosphatases PP1 and PP2A contribute to the dynamic phosphorylation landscape of SRSF1 [92,98,99,100]. In addition to the PTMs of the RS domain, SRSF1 is also methylated at multiple Arg residues within the RRM region, which modulates SRSF1 nuclear–cytoplasmic shuttling and its functions in each cellular compartment [101].

SRSF1 is also known to be recruited to transcriptionally active sites with a less condensed chromatin state, where it is associated with the proteins Psip1/Ledgf, suggesting that SRSF1 might also have a function in transcription regulation [102]. A DNA binding capability of SRSF1 in association with the 7SK snRNP to the promoter regions was described; however, the precise mechanism by which SRSF1 modulates cellular transcription remains poorly understood [60,103]. This property is crucial for understanding the virus–host interactions as SRSF1 has been shown, for example, to inhibit the promoter activity of JC polyomavirus (JCPyV) at elevated concentrations [104].

### 2.3. Heterogeneous Nuclear Ribonucleoproteins

**Figure 4:**
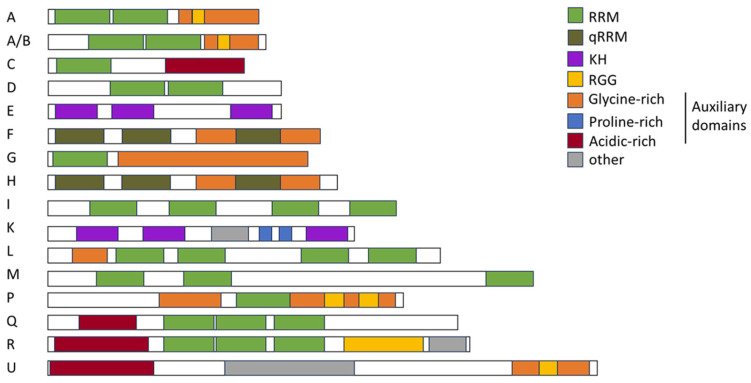
Schematic drawing of the structural landscape and domain architecture of hnRNPs. hnRNP families A, A/B, C, D, E, F, G, H, I, J, K, L, M, P, Q, R, and U are depicted. RNA recognition motif (RRM), quasi-RNA recognition motif (qRRM), K-homology (KH), arginine–glycine–glycine (RGG), as well as glycine-rich, proline-rich, and acidic-rich domains are depicted in the indicated colors. Illustration was adapted from [106].

The second-largest RBP protein family consists of heterogenous nuclear ribonucleoproteins (hnRNPs) [105,106,107]. They constitute a diverse family of proteins intricately involved in the regulation of RNA metabolism. The hnRNPs exhibit a modular architecture characterized by the presence of RNA recognition motifs serving as the primary interface for RNA binding, maintaining their sequence-specific binding to the *cis*-regulatory elements on mRNAs and pre-mRNAs (Figure 4). The RRM mediates the interactions with (pre)-mRNA [108] and further RNA binding, while increasing specificity is achieved by the quasi-RRM (qRRM) [109,110]. The hnRNPs may include additional RNA-binding domains, such as a glycine-rich RGG box and a K-homology (KH) domain [111]. Despite their RNA-binding capacity, hnRNPs are capable of binding DNA. hnRNP U is able to bind AT-rich DNA elements with their C-terminal arginine- and glycine-rich domains [112] and therefore influences the chromatin organization [113]. Other members of the hnRNP family, like hnRNP A1, A2/B1, D, E, and F/H, have been described to bind DNA telomeres and regulate telomerase activity, directly impairing the telomere length [114,115]. Furthermore, recent studies suggest that hnRNP A2/B1 is a sensor for nuclear DNA. As a homodimer, hnRNP A2/B1 can bind ssDNA and dsDNA and relocate to the cytoplasm, where it stimulates the cell innate viral immune response and interferon signaling [116].

The RGG box is considered as an auxiliary domain, forming homologous and heterologous interactions with hnRNPs and other proteins [117]. The auxiliary domains mediate protein–protein-interactions and nuclear localization [118] and often consist of proline-, glycine-, or acid-rich domains [119]. The elemental building blocks within a family are therefore identical, but they exhibit variations within their sequence. Here, the combination of these domains defines a large diversity of functionality of hnRNPs [106].

Through this diversity in composition and functionality, the hnRNP family can be grouped into subfamilies (Figure 4) hnRNP A/B (A0, A1, A2B1, and A3), hnRNP C (C1 and C2), hnRNP D and hnRNP E (E1, E2, E3, and E4), hnRNP F/H (F and H1/H2), hnRNP G and hnRNP I/L (I and L), hnRNP K and hnRNP M/Q (M and Q), and hnRNP P2 and hnRNP R/U (R and U) [106]. Both the SRSF and hnRNP protein families are the main types of *trans*-acting splicing factors [120]. In particular, the subfamilies hnRNP A/B, hnRNP D, and hnRNP F/H have been shown to be involved in splicing regulation [43,121,122] (see Section 5). Here, in a genome-wide analysis of hnRNP binding to HIV-1 RNA, a key role for hnRNP H1 in alternative splicing was revealed [123]. In addition, their ability to form homo- or heteromers further increases their functional diversity [124]. One example is hnRNP A2/B1, a monomeric protein that functions as an m6A reader, pivotal in the RNA transport, processing, and mRNA splicing mechanisms. Upon binding to viral DNA, hnRNP A2/B1 undergoes dimerization and demethylation, facilitating its translocation from the nucleus to the cytoplasm, thereby activating the TBK1–IRF3 pathway and stimulating the innate immune response against viral pathogens [125]. Other functions of hnRNPs include RNA editing, mRNA stabilization, nuclear export, translation, and telomere biogenesis [106,126,127]. Furthermore, a recent study suggests that hnRNP K can initiate the translation of retroviral mRNAs in a cap-independent manner using an internal ribosome entry site (IRES) [128]. Hereby, hnRNP K acts as an IRES-trans-acting factor [128]. While low tissue specificity for hnRNP proteins is predicted [51], hnRNP H2 and hnRNP UL1 show cell type-specific enrichment, with high abundance in syncytio-trophoblasts and dendritic cells, respectively. Classified as low-tissue-specific, the members of the hnRNP A/B subfamily exhibit high expression across nearly all tissues. An exception, however, is hnRNP A0, which is expressed in only low to medium quantities in most tissues but at high concentrations in the neuronal cells in the cerebral cortex and caudate, the stomach, the testis, and the placenta [52].

Comparable to SRSFs, hnRNPs also underlie PTMs, including phosphorylation, methylation, acetylation, and ubiquitination as a significantly higher amount of PTM sites have been identified in hnRNPs versus all other RBPs, suggesting a strong PTM-dependent regulation [105,129,130,131]. Arginine residues, in particular, are known to undergo methylation, a modification that plays a crucial role in nuclear export and can influence the ability of hnRNPs to bind nucleic acids by altering the charge of arginine. It has been predicted that hnRNPs account for approximately 65% of all methylated arginines in the nucleus, suggesting a central regulatory role in hnRNP function.

### 2.4. Heterogenous Nuclear Ribonucleoprotein A0

hnRNP A0 is part of the hnRNP A/B family [106], but, in contrast to its other family members, hnRNP A1, hnRNP A2/B1, and hnRNP A3, it has not been previously described as a cellular and HIV-1 splicing factor [43,121,122,131,132]. In fact, hnRNP A0 is the least-studied member of the hnRNP A/B family, and therefore not much is known about its specific role. Nonetheless, hnRNP A0 was described to play a role in the post-transcriptional mRNA regulation of different transcription factors [133]. It has also been shown that hnRNP A0 can regulate cell cycle arrest through the interaction with the checkpoint kinase MK-2 and can lead to cellular resistance against the DNA damage caused by chemotherapy [134]. Furthermore, it is linked to cancer cell growth in different cancer types, such as lung or gastrointestinal cancers [134,135]. There has been a consensus binding sequence identified for hnRNP A0, which consists of a pentamer AUUUA [133]. Generally, hnRNP A0 seems to bind the 3′UTR of mRNAs to adenylate–uridylate (AU)-rich elements (AREs) [136,137,138]. Of note, HIV-1 contains many AREs dispersed throughout its genome, including its 3′-LTR [139]. As with the other hnRNP A/Bs [131], its binding affinity is also regulated through PTM and phosphorylation. Furthermore, hnRNP A0 is phosphorylated at Ser-84 through MAPKAP-K2 [133], and other potential phosphorylation sites have been predicted [130] (http://PTM-RBP-ATLAS.igb.uci.edu; accessed on 30 April 2024).

## 3. Cellular Splicing Factors SRSFs and hnRNPs Are Involved in Immunity and Underlie Interferon-Mediated Regulation

Since the discovery of interferons (IFNs) and their antiviral capacity, numerous studies have focused on induced genes known as interferon-stimulated genes (ISGs) [140]. Depending on their binding affinity towards different receptors and sequence analogy, IFNs are categorized into types I–III IFNs. Upon IFN stimulation, the cellular gene expression patterns shift, leading to the expression of numerous genes controlled by interferon-sensitive response elements (ISRE) and gamma-interferon activation sites (GAS). ISREs are primarily induced by type I and type III IFNs (IFNα, IFNβ, IFNκ, IFNε, IFNω, and IFNλ), while the GAS elements are primarily induced by type II IFN, which is IFNγ. Almost all cells express the IFN-I and IFN-II receptors and are capable of expressing IFN-I, but IFN-II is produced only by antigen-activated T lymphocytes and cytokine-activated group 1 innate lymphoid cells [141,142,143].

Human interferons exhibit distinct structural features, receptor interactions, signaling pathways, and biological functions. While IFNβ is encoded by a single gene, IFNα represents a family of 13 subtypes encoded by individual genes located on chromosome 9, while 12 individual IFNα subtypes (1, 2a, 2b, 4a, 4b, 5, 6, 7, 8, 10, 14, and 21) are expressed [144,145]. They are highly conserved, with an amino acid (aa) sequence similarity between 75 and 99% [145]. Both IFNα and IFNβ bind to the same receptor complex, composed of the IFNAR1 and IFNAR2 subunits; however, despite this high similarity, the differences between the subtypes in terms of HIV infection and immunity are substantial [146,147]. Upon the binding of IFN-I, the kinases JAK1 and TYK2 are recruited, which results in the subsequent phosphorylation of the transcription factors STAT1 and STAT2. Upon phosphorylation, IRF9 is recruited and the ISGF3 complex is formed, which shuttles into the nucleus, activating ISREs, and thereby inducing the expression of antiviral genes, including host restriction factors [148]. In addition, the family of IFN type III (IFN-III) consisting of IFNλ subtypes 1–4 [149,150,151] primarily exerts antiviral effects at barrier surfaces such as the respiratory or gastrointestinal tracts [152]. The heteromeric IFN-III receptor complex is formed by interferon-lambda-receptor 1 (INFLR1) and IL-10R2 [153,154,155]. Similar to IFN-I, the IFN-III receptor complex is accompanied by JAK1 and TYK2 and thereby uses a similar signaling cascade via STAT1 and STAT2 [156]. The IFN-II receptor complex consists of the two subunits interferon-gamma-receptor (IFNGR) 1 and IFNGR2. Following the interaction with IFNγ, the kinases JAK1 and JAK2 undergo phosphorylation, leading to the subsequent phosphorylation of two STAT1 proteins. These phosphorylated STAT1 proteins then form homodimers, translocate into the nucleus, and activate GAS.

IFNα2, which is the only IFN clinically used against hepatitis B virus (HBV) and hepatitis E virus (HEV), has been extensively studied. In HIV-1-infected humanized mice, IFNα2 showed only minor protective effects, whereas IFNα14, delivered at the same clinical dose, showed very potent antiretroviral activity [157]. In an acute infection model, a stronger inhibition of viral replication was observed in mice treated with IFNα14 compared with IFNα2. IFNα14 proved to be vastly more potent than IFNα2 as the HIV-1 p24 and RNA levels were suppressed below the detection limit when administered as a post-exposure prophylaxis (PEP). In agreement, the IFNα14-treated cells demonstrated significantly higher ISG expression, including HIV-1 restriction factors tetherin or MX2, compared to IFNα2-treated cells. Differences in ISG expression were also observed in HIV-1-infected lamina propria mononuclear cells (LPMCs) stimulated with IFNα subtypes. IFNα8 and IFNα14 induced both ISGs to a greater extent than IFNα2 and, in particular, IFNα1. The IFNs that induced antiviral ISGs more strongly also exhibited a greater antiviral effect, as determined by the HIV-1 p24 levels and infectivity testing in TZM-bl reporter cells [158]. In agreement, the in vitro stimulation of PBMCs with IFNα subtypes also identified IFNα14 as the strongest inducer of HIV-1 restriction factors as it strongly upregulates the expression of MX2, tetherin, and Trim22 [159]. In line with a study categorizing IFNs based on their activity levels into low, intermediate, and high, IFNα14 was classified among the highly active IFNs and is notably potent against HIV-1 due to its induction of robust host restriction factors [147,160].

In recent years, alongside the detailed study of various IFNα subtypes, there has been increasing research on interferon-repressed genes (IRepGs). Initially, using a method to analyze the short-term changes in RNA synthesis, Doelken et al. found a new subset of genes that were downregulated 30–60 min following IFNγ treatment [161]. Generally, the identified genes were involved in gene expression, apoptosis and cellular proliferation, growth, and development. In a follow-up study using the labeling of newly transcribed RNA by 4sU in combination with translational arrest using cycloheximide during IFN treatment, the authors identified STAT1 as a potential key transcription factor of more than half of the identified IRepGs [162]. They speculate a dual role of STAT1 by the stimulation of ISGs by STAT1 homodimers and expression of other genes by other STAT1-containing complexes. The observed repression could thereby be explained by the deprivation of the other STAT1-containing complexes upon the IFNγ-induced phosphorylation of STAT1 homodimers. Furthermore, the authors observed that the IRepGs-encoded transcripts were rather short-lived, with a median half-life of t_1/2_ = 90 min. They concluded that this would be indicative of transcription factors or genes involved in cell signaling. In a different study, using a proteome analysis via the mass spectrometry of IFNα- and IFNγ-treated cells, the host-dependency factors (HDFs) for HIV-1, which were previously identified using siRNA screenings [163,164,165], were identified as IFN-regulated [166]. Among those, several RNA-binding proteins (RBPs) were identified, in agreement with the hypothesis that transcription factors are among the IRepGs.

In 2022, we demonstrated that the SRSF1 expression levels are significantly lower in abundance in the LPMCs and PBMCs obtained from people with HIV-1 (PWH) compared to cells from PWH receiving ART or people without HIV [46]. The cells obtained from acute and chronic PWH had elevated *ISG15* levels, indicating elevated IFN signaling and identifying SRSF1 as an IRepG. A change in the expressed subset of the IFNα subtypes and an overall elevated IFNα-expression in PWH were already observed in previous studies [147,159,167]. We additionally observed a significant correlation between the *ISG15* induction and *SRSF1* repression in HIV-1 acute PWH. When analyzing the different IFNα subtypes, we observed IFNα14 as the strongest in its capacity to repress *SRSF1* expression, while IFNα2 did not cause a strong repression. Treating differentiated THP-1 cells with both IFNs revealed IFN-specific kinetics, with IFNα2 repressing the *SRSF1* expression only 12 h following the treatment while IFNα14 caused significantly decreased expression from 12 to 48 h. A subsequent protein analysis via Western blot also showed a stronger decrease for the IFNα14-treated cells. Notably, the IFNγ-treatment of THP-1 cells induced the *ISG15* mRNA expression levels to comparable but overall weaker levels than IFNα2 and IFNα14. Additionally, the *SRSF1* expression kinetic differed between IFN-III and IFN-I. The IFNγ-treated cells showed repressed expression levels at 8 h following the treatment; from there on, the expression levels continuously increased until 1.5-fold 48 h following the treatment. This interferon-regulated alteration in the SRSF1 expression levels significantly affected the HIV-1 replication. Mechanistic studies using transiently transfected HEK293T cells with siRNA (knockdown) or an SRSF1-expressing plasmid (overexpression) and the proviral vector pNL4-3 revealed increased HIV-1 particle formation and infectivity under low SRSF1 levels, while a strong decrease was observed under high SRSF1 levels [46].

High SRSF1 expression levels have also been correlated with the transcriptional inhibition of other viruses. The human polyomavirus JC virus (JCPyV), listed as an AIDS-defining disease, can become active in immunocompromised individuals, causing progressive multifocal leukoencephalopathy (PML) [168,169]. It was shown that a soluble immune mediator secreted by PBMCs, which has not been further defined, regulates neuronal SRSF1 expression. A disturbance in the neuroimmune signaling and SRSF1 in immunocompromised individuals could result in the expression of JCPyV early genes, which might trigger productive replication, leading to the development of PML. The authors further showed that a prolonged 50% decreased expression of SRSF1 resulted in the expression of JCPyV early genes independent of the presence or absence of soluble immune mediators. Additionally, it was shown that SRSF1 can directly bind the promoter of the JCPyV and thus repress gene expression [104]. In agreement, SRSF1 expression is inhibited by the viral LT protein and the RBP Pur-α. Furthermore, SRSF1 expression can be upregulated by the mitochondrial proteins TIM22 and TIM29 upon HBV infection, which causes the SRSF1-mediated suppression of the HBV core promoter [170].

In addition to IFN-I, the signaling pathways for TNF-α play a central role in the immune response, particularly in the regulation of inflammation and antiviral defense mechanisms. Although TNF-α does not directly activate the JAK-STAT pathway, it can trigger secondary signaling cascades that lead to the activation of STAT proteins, for example, by inducing interferon production and secretion in certain cell types. Additionally, type I interferons and TNF-α overlap in the activation of NF-κB, MAPKs, and the induction of apoptosis. Indeed, this fact could also be associated with interferon-repressed genes as the SRSF1 expression was shown to be lower in the muscle biopsy samples from patients with inflammatory myopathy [171]. The authors linked the downregulation to a potential TNFα-mediated pathway, which hardens the hypothesis that SRSF1 underlies immune signaling regulation. However, as described above, SRSF1 itself is involved in numerous immunomodulatory functions by exerting pleiotropic effects on transcription, alternative splicing, mRNA stability, mRNA export, translation, and even microRNA processing [102]. Interestingly, the translational repression of TNFα mRNA is facilitated by the ARE located in the 3′-untranslated region [172], which is a binding site of the hnRNPA/B family [131], including hnRNP A0, which is important for the post-transcriptional regulation of TNFα mRNA [133].

In our recent study, the RBP hnRNP A0 was found to be repressed the strongest, of the hnRNP A/B family, by IFNα14 [47]. Interestingly, in Jurkat T-cells and monocyte-derived macrophages (MDMs), we observed a significant overexpression of *hnRNP A0* 48 h following the treatment and following an initial repression, observed between 4 and 24 h. Blocking IFN signaling with the JAK1/2 inhibitor Ruxolitinib, we demonstrated that the repression of hnRNP A0 depends on either of these kinases, implicating STAT1 involvement, as described earlier. Of note, STAT1 is also labeled as a transcription factor of hnRNP A0 by the ENCODE Transcription Factor Targets data set [173,174]. The regulation of hnRNP A0 by interferon significantly impacts HIV-1 replication. Follow-up mechanistic studies in transiently transfected HEK293T and infected Jurkat T-cells demonstrated beneficial effects for HIV-1 under low hnRNP A0 levels. This resulted in increased transcriptional activity and export of unspliced mRNA, leading to enhanced viral replication, higher viral titers, and increased infectivity. Conversely, high hnRNP A0 levels strongly inhibited transcription, unspliced mRNA export, and programmed ribosomal frameshifting, substantially decreasing the HIV-1 replication capacity [47]. Finally, we observed lower hnRNP A0 expression levels in acute and chronic PWH compared to people without HIV. In both groups, we also observed higher *ISG15* expression levels, confirming hnRNP A0 as an IFN-I-repressed cellular splicing factor. Interestingly, co-infections exacerbating the inflammatory state exhibited elevated expression and altered HIV-1 splicing, correlating with an augmented HIV-1 reservoir size in cART-treated PWH [175,176].

The extent to which HIV-1 exploits the inflammatory conditions and IRepGs for replication efficiency versus IRepGs serving as a cell-specific adaptive mechanism potentially creating unfavorable conditions for viral replication warrants further investigation. The initial repression of SRSF1 and hnRNP A0, however, is advantageous for HIV-1 in the post-integration stages. Consequently, in the following chapters, we will explore the impact of interferon-regulated hnRNP and SRSFs on the key steps of viral replication, with a focus on HIV-1.

## 4. Impact of Interferon-Regulated Splicing Factors on LTR Transcription

As described in Section 3, the members of the SRSF and hnRNP families have been identified as IFN-repressed splicing factors [46,47]. We recently investigated the IFN-mediated regulation and demonstrated early repression of SRSF1 and hnRNP A0 following IFN treatment [46,47]. In addition, the extensively studied cellular host splicing factor hnRNP A1, which is important for balanced HIV-1 gene expression, also exhibited reduced mRNA expression levels in THP-1 cells treated with IFNα14 [47]. A common feature among all these host factors is their ability to additionally modulate the transcriptional activity of HIV-1. At elevated concentrations, they led to a reduction in the overall viral mRNA abundance, whereas, at lower concentrations, they resulted in an increase in the viral mRNA levels, with a minimal impact on the mRNA stability [46,47,94]. Notably, the IFNα14 treatment in THP-1 cells did not alter the hnRNP A2B1 expression levels [47], consistent with the requirement for a specific range of hnRNP A2B1 levels for optimal HIV-1 replication. Both elevated and decreased expression levels of hnRNP A2B1 have been demonstrated to impair HIV-1 transcriptional activity [94].

HIV-1 infection per se modulates the abundance of RBPs in various ways. The expression of Tat in the nucleolus of T-cells, for example, causes a significant depletion of hnRNP A2B1 [177]. The hnRNP A1 expression increase upon infection and its nucleocytoplasmic trafficking was impaired in infected T-cells. However, no changes in nucleocytoplasmic shuttling were observed in HIV-1-infected MDMs over 6 weeks, suggesting cell-type-specific interactions. Additionally, the expression levels of hnRNP A1, A2/B1, and H were repressed in the first week following the infection, following a steady increase to slightly higher expression levels compared to mock-infected MDMs [178]. In the first week following HIV-1 infection, the SRSF2 expression was significantly increased in H9 cells [179] and MDMs [178]. At later time points, however, the expression levels were restored to normal amounts.

**Figure 5:**
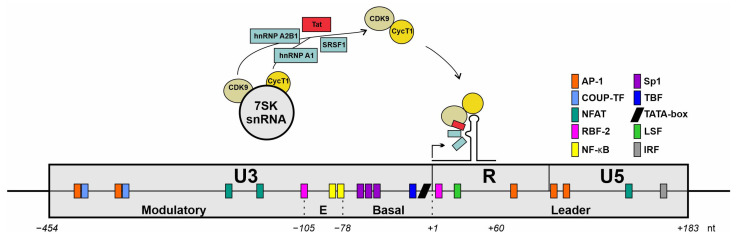
Schematic representation of an HIV-1 long terminal repeat (LTR) architecture. Interactions with cellular transcription factors are illustrated. Schematic drawing of the LTR of the HIV-1 genome with nucleotide positions relative to the transcriptional start site (+1). The promotor comprises three main regions: U3, R, and U5 encompass specific functional domains; the U3 region contains the modulatory segment; the R region includes the TAR loop structure that is essential for HIV-1 transcriptional regulation. Colored boxes highlight potential binding sites for cellular transcription factors. Figure adapted from [206].

Noteworthily, RBPs exert multifaceted influences on HIV-1 transcription as they participate in almost all the stages of gene expression, including transcription, alternative splicing, mRNA trafficking, and translation. SRSF1, for example, can directly affect HIV-1 transcription by competing with Tat for the overlapping sequences within the TAR within the LTR [180] or indirectly affect Tat-mediated transcription by also competing with the binding sites of the 7SK particle [180] (Figure 5). When HIV-1 Tat binds to the 7SK snRNP, it induces the release of p-TEFb [181], which can then bind to the TAR of the LTR. This interaction is essential for the Tat-mediated transcriptional elongation of the RNAPII [10,182,183,184,185,186]. When bound to the 7SK snRNP, SRSF1 renders P-TEFb largely inactive. It was shown that, under high levels, SRSF1 competes with Tat for binding onto TAR, which decreases the viral transcription [187]. Hence, the initial repression of SRSF1 following IFN stimulation would facilitate Tat-mediated trans-activation. When SRSF1 binds to nascent RNA transcripts, it destabilizes the 7SK snRNP, leading to the dissociation and activation of P-TEFb, consequently facilitating transcription elongation [102]. Notably, hnRNP A1 has also been described as a reversible part of the 7SK SRNP [188], and the knockdown of hnRNP A1 attenuated the dissociation of P-TEFb from the 7SK snRNP, elongating its active phase [189]. Furthermore, hnRNP A1 binds to the SL3 domain of the 7SK SRNP, which contains a putative high-affinity hnRNP A1 binding sequence (260-UAGGGU-265) [189,190]. Although multiple studies have demonstrated the binding of hnRNP A0 and A1 to identical AREs, with both proteins notably binding the pentamer “AUUUA” [107,133,137], an interaction of hnRNP A0 and the 7SK snRNP has not been observed thus far. Low levels of hnRNP A0 likely enhance the HIV-1 Tat-mediated transcription, while high levels decrease the transcriptional activity. Moreover, in anti-hnRNP A0 siRNA-transfected luciferase reporter cells lacking Tat, a reduced LTR activity was observed [47], which was also reported in the context of SRSF1 [180].

Another level of transcriptional modulation by RBPs is by affecting the splicing efficiency of the eight Tat isoforms [11]. The Tat protein is encoded by two exons, with exon 1 encompassing Tat aa 1–72 and exon 2 spanning Tat aa 73–101. Notably, a truncated yet functionally trans-activating Tat protein (Tat 5–8, 4 kb mRNA class), solely comprising the initial exon, was described [11,191,192]. However, the clinical isolates almost exclusively express full-length Tat (Tat 1–4, intronless 2 kb mRNA class) [11,192]. The splicing of *tat 1*–4 coding transcripts is facilitated by the co-occurring splicing of both exons using the splice donor D1 to splice acceptor A3 and subsequent splicing from D4 to A7. To facilitate this process, multiple SREs, which act in concert, are required. Exonic-splicing tat enhancer (ESE_tat_) [193,194], ESE2 [126,195], exonic-splicing-silencer2 (ESS2) [196,197,198], and ESS2p [199] are bound by multiple RBPs like hnRNP A1, SRSF2, hnRNP H, and SRSF6 (see Section 5; reviewed in [43]). High levels of SRSF2 were shown to increase the *tat* mRNA in transfected HEK293T cells [95]. Moreover, HIV-1 infection in MDMs resulted in elevated expression of SRSF2, which might in turn facilitate HIV-1 transcription by elevating *tat* mRNA. Notably, the expression levels of hnRNP A1, which negatively influences *tat* mRNA splicing by binding to an ISS [200], thereby inhibiting the intron splicing of the *tat* mRNA, were concomitantly decreased [178].

It is worth mentioning that binding sequences are not exclusively used by a single RBP but are often bound by multiple other RBPs [201,202,203]. An illustrative example is provided by hnRNP A0 and hnRNP A1, which share an identical binding motif “AUUUA” [107,133]. However, this does not imply that these RBPs necessarily perform identical physiological functions upon binding the same sequence [201]. Particularly, the drosophila-related homologs of the hnRNP A/B family bound overlapping target sequences while still explicitly binding to distinct target RNAs [201,204]. By binding to AREs, hnRNPs can affect post-transcriptional steps like translation or mRNA degradation [131]. As described above, hnRNP A0 modulates the expression of the TNFα mRNA by binding to its ARE [133]. Here, TNFα was shown to synergistically boost the HIV-1 transcription in the early stages of infection by the TNF-mediated translocation of NF-κB [205].

There is accumulating evidence that RBPs are also involved in HIV-1 latency in a multifactorial manner. It was shown that the *tat* expression levels declined in MDMs after an initial spike early following the infection [207]. The authors of this study linked the decrease in *tat* expression to changes in the expression of SRSF2 and hnRNP A1 [178], which are crucially involved in the *tat*-mRNA splicing process, as discussed above. Additionally, a transcriptome analysis using the bulk RNA-Seq of three different primary CD4^+^ T-cell models, used to study HIV-1 latency, revealed alternative splicing as pronounced and the most consistent mechanism of latent HIV infection. A common set of 234 differently regulated cellular genes were identified while comparing unstimulated and stimulated CD4+ T-cells, albeit the RBPs of the SRSF and hnRNP group were not among the stimulated vs. unstimulated common set as none were identified in a resting-cell model, and several SRSFs and hnRNPs were identified in the CD4+ T cells obtained from PWH receiving ART and in the infected CD4+ T cells (hnRNP A1, A2B1, C, F, L, M, R/SRSF1, 5, 8, and 9) [208]. Notably, hnRNP A2B1 was described to modulate the HIV-1 latency by unfolding epigenetic regulatory G-quadruplexes within the HIV-1 LTR, thereby activating HIV-1 transcription [209]. In another study analyzing HIV-1 gene expression, the modulation of serine–arginine kinase (SR kinase) expression via shRNA or kinase activity using pyridazine derivatives revealed distinct features. The depletion of CLK1 increased the HIV-1 LTR activity and boosted the activity of latency-reversal agents (LRAs), while CLK2 affected the steps following transcriptional initiation. Finally, the authors hypothesize that the modulation of SR kinases might help in block-and-lock or shock-and-kill approaches as CLK1 inhibition would facilitate latency reversal and, on the other hand, CLK2 inhibition would be beneficial for latency [210]. A blockade of the A7 splice acceptor site, crucial for the formation of multiple spliced mRNAs, including tat mRNA, was also discussed as a strategy to achieve a locked state of latency [211].

Considering the diverse array of activities exhibited by RBPs, it is unsurprising that they participate in multiple mechanisms that directly or indirectly influence HIV-1 transcription. As research progresses, the catalog of identified interferon-regulated HIV-1 regulating host factors will expand. Simultaneously, the repertoire of processes through which these RBPs engage in transcriptional regulation, either directly or indirectly, will also increase. However, deciphering the intricate network of RBPs to attribute indirect effects to specific proteins is challenging due to their heterogeneous and pleiotropic nature and intricate involvement in the multifaceted functions within a network of other RBPs.

## 5. Impact of Interferon-Regulated Splicing Factors on HIV-1 Alternative Splicing

The determination of splice site pairing relies on two successive processes: recognition and pairing. Within this context, either step is tightly regulated by both the intrinsic strength of the 5′ and 3′ splice sites as well as by *trans*-acting cellular splicing factors bound to *cis*-acting splicing regulatory elements (SREs) (reviewed in [43]). The large landscape of SREs, which provides binding sites for regulatory proteins such as SRSF and hnRNPs, implies stringent regulation. Indeed, both the SRSF and hnRNP protein families have been described as main *trans*-acting splicing factors [120]. The spatial relationship between an SRE and a splice site dictates its function as either an enhancer or a silencer in a rigorously position-dependent manner [212,213]. Specifically, SRSF proteins promote splicing when located within an exonic context, upstream of the 5′ splice site (ss), while exerting a repressive effect when situated in an intronic position downstream of the 5′ ss. Conversely, hnRNPs stimulate splicing when positioned in an intron but exhibit suppressive activity upon recruitment to an exon [212,213]. Also, variability in the RNA conformation influences the selection of splice sites and impacts the expression of viral genes [214]. Together, these factors constitute the ‘splicing code’ [215,216], reflecting the complexities of splicing control. The mutational disruption the of *cis*-regulatory elements through, e.g., substitution, can substantially impair the HIV-1 splicing and replication efficiency [193,217,218,219,220,221]. Significantly, the expression levels of splicing factors, which might be tissue specifically different per se, may critically influence the splicing outcome following IFN-induced STAT1/STAT2 signaling [46,47] or TNFα treatment [171], which were both shown to temporally downregulate the expression levels of SRSF and hnRNP levels, particularly SRSF1 and hnRNP A0.

**Figure 6:**
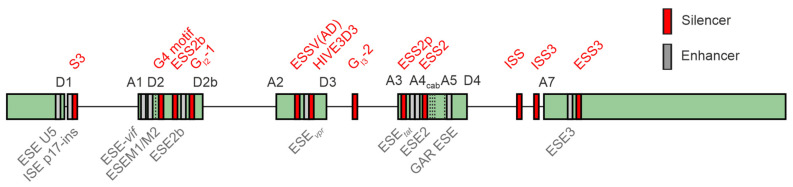
Splicing-associated *cis*-regulatory elements in the HIV-1 genome. The arrangement of SREs (splicing regulatory elements) within the HIV-1 genome is illustrated. Exons are represented by gray boxes, whereas introns are depicted as black lines. Splicing enhancers are highlighted in green, while splicing silencers are indicated in red. Illustration was modified according to [43].

The HV-1 4 kb mRNA class predominantly undergoes splicing at the primary splice donor site (D1) towards one of the acceptor sites located within the central cluster, while 2 kb mRNAs are subjected to additional splicing events originating from the central splice donor (D4) towards the terminal acceptor site (A7). The significance of the 1 kb mRNA transcript recently identified in an RNA sequencing analysis for viral replication [44] remains uncertain. The equilibrium of all the HIV-1 mRNA isoforms is tightly regulated by a complex network of *cis*-regulatory elements bound by *trans*-acting cellular host dependency factors (Figure 6). The HIV-1 leader exon 1 harbors two splicing enhancers, ESE U5 [222], ISE p17-ins [222,223], and the negative regulator of D1 recognition S3 [224]. Leader exons 2 and 2b, respectively, both important for *vif* formation, are tightly regulated by a network of multiple splicing enhancers, including ESE-*vif* [219], ESEM1/M2 [79], G4 motif [219], ESS2b [225], ESE2b [225], and the intronic G-rich element G_I2_-1 [217]. Balanced levels of leader exon 3 inclusion and thus *vpr* formation are regulated by ESSV (AD) [221,226,227,228], ESE_vpr_ [229], HIVE3D3 [230], and the deep intronic G run G_I3_-2 [218]. Exon 4 inclusion is tightly regulated by ESS2p [199], ESEtat [193], ESE2 [126,195], ESS2 [196,197,198], and the GAR ESE element [78]. The terminal exon begins with HIV-1 splice acceptor 7, ISS [231], ESE3 [93], and ESS3 [93,232,233]. A comprehensive overview of the known HIV-1 SREs and their impact on viral replication is presented in [12,43,234].

Synonymous mutations within viral genomes that lack amino acid alterations can exert an influence on diverse biological processes, including the transcription, RNA structure, splicing, translation, and microRNA targeting. The effect of synonymous genome recoding on splicing was investigated in the context of the HIV-1 *env* gene to explore how differential codon use affected the HIV-1 Env protein expression and virus viability. Here, the alteration of a single codon located in the gp41 coding region, which was included in an intronic splicing silencer SRE, completely abolished the virus replication and Env expression. The computational analyses revealed a severe disruption in the SRE RNA secondary structure due to this synonymous mutation. In contrast, the overall alteration of the codon use in the gp120 region outside the known SRE by introducing an increased number of CpG dinucleotides did not significantly affect the Env expression or virus viability. These findings suggest that synonymous recoding can impact the viral phenotype and alter the virus biology by affecting critical virus mRNA secondary structures implicated in RNA splicing and protein expression [235]. Also, drug resistance-associated mutations like R263K might result in the altered HIV-1 mRNA splicing, e.g., of exons 2 and 2b, determining the *vif* mRNA formation. Here, a synonymous variation in the mutation with a similar bioinformatically predicted impact on SREs was shown to have comparable ablative effects on the recognition of D2b as its non-synonymous counterpart [236].

Vif is essential for HIV-1 replication in natural target cells as it counteracts the host restriction factor APOBEC. Within target T lymphocytes, HIV-1 encounters retroviral restriction factors such as APOBEC3G (A3G). This interaction is counteracted by the HIV-1 accessory protein Vif, which is encoded by intron-containing viral RNAs generated by splicing at splice acceptor A1 but lacking splicing at splice donor D2. As a result, a large downstream intron is retained. The extent of the activation of 3′ss A1 and repression of D2 determine the levels of vif mRNA and the ability to evade A3G-mediated antiviral effects [217]. The use of 3′ss A1 can be enhanced or repressed by splicing the regulatory elements that control the recognition of downstream 5′ss D2. It has been shown that the single-nucleotide variations naturally occurring within the SA1D2 proximal region of the HIV-1 genome can modify the Vif expression levels by the alteration of SREs, impacting the inversely correlated levels of *vif*/*vpr* mRNAs [237]. We and others demonstrated that infrequently utilized SD2b [11,238] may facilitate *vif* formation and counteract APOBEC3G [218]. Furthermore, mutant strains deficient in adequate *vif* levels acquire compensatory mutations that enhance the SD2b utilization, underscoring the significance of the SD2b nucleotide sequence in the *vif*-mRNA production pertinent to HIV-1 adaptation. This highlights the mutual antagonism between Vif and APOBEC3 proteins in HIV-1 adaptation, evolution, and survival [239].

SREs offering binding sites for SR proteins and hnRNPs highly influence the RNA processing steps within the *gag* coding region [240]. It was found that this region is regulating viral mRNA processing and infectivity through a specific nucleotide bias. It was shown that increasing the GC content by synonymous mutations can activate a cryptic splice donor site, which causes a disruption in the balanced viral splicing pattern, ultimately affecting infectivity [241]. This underscores the importance of the nucleotide composition in this region, which appears to guide RBPs in determining the RNAs’ fate. Here, the overexpression of SRSF9 was shown to decrease the *gag* mRNA levels in a dose-dependent manner. Due to the sequence and structural similarities between SRSF9 and SRSF1, the overexpression of SRSF1 was shown to disrupt the balance of the alternative splicing of viral mRNA and inhibit *gag* in a similar manner [95,242].

Several mechanisms have been described for interferon-regulated SRSF1 regarding its influence on alternative HIV-1 RNA splicing. Primarily, the purine-rich ESE guanosine-adenosine-rich (GAR) element significantly regulates the 3′ss central cluster in a bidirectional manner. The use of the enhancer that is localized between A5 and D4 is important for the formation of *rev* and *nef* mRNAs. Its activation causes the recognition of the 3′ss and 5′ss and the inclusion of the internal exons 4c, 4a, 4b, and 5, which is achieved by the binding of SRSF1 and SRSF5 to three distinct binding sites [93]. The proposed mechanism is promoting the U1 snRNP binding and thus D4 use [78], while the binding of the U1 snRNA induces cross-exon interactions and splice site pairing [243,244]. The recognition of splice acceptor A1, which is important for *vif*-mRNA formation, has been reported to underly the regulation by three SREs in exon 2, including the SRSF1-bound ESE M1/M2, which synergistically promotes D2 recognition and exon inclusion [79]. Furthermore, the SRSF1-bound ESE3 enhancer, positioned downstream of A7, has been demonstrated to promote the stabilization of the U2-associated U2AF65 subunit at the splice acceptor site [79].

In contrast to the well-characterized SRSF1, relatively little is known so far about the characteristics of hnRNP A0 in cellular and HIV-1 alternative splicing. Despite sharing a binding motif with hnRNP A1, hnRNP A0 may exhibit substantial functional differences. Further systematic functional investigations are necessary to mechanistically understand the splicing regulatory effect observed on HIV-1 splicing upon the overexpression of hnRNP A0.

## 6. Impact of Interferon-Regulated Splicing Factors on mRNA Trafficking

The export of HIV-1 mRNAs is a crucial step for viral replication, with distinct mechanisms employed for different classes of transcripts. The intronless, i.e., fully spliced, 2 kb class utilizes the nuclear RNA export factor 1 (NXF1)-mediated export pathway for nucleocytoplasmic shuttling, similar to the majority of host cellular mRNAs. In contrast, the 4 kb and 9 kb classes of intron-containing and unspliced transcripts primarily rely on the more specialized chromosomal region maintenance 1 (CRM1)-mediated export pathway facilitated by the re-imported viral protein Rev binding to the RRE [245]. This differential reliance on export mechanisms underscores how HIV-1 strategically modulates its gene expression post-transcriptionally to control the production of the essential viral components in a dynamic and timely manner.

NXF1 plays a crucial role in the export of eukaryotic and viral mRNA from the nucleus to the cytoplasm. NXF1 interacts with both mRNA molecules and various nucleoporins (Nups), particularly within the peripheral ring of the nuclear pore complex (NPC). The NXF1 export protein is generally able to bind RNA through an N-terminal arginine-rich RBD that shows no direct sequence specificity and thus is capable of interacting with a variety of RNA substrates. The interactions, however, are weak and unspecific [246]. Thus, NXF1 requires the adaptors to allow for efficient interactions with both eukaryotic and viral mRNA [247]. Here, the Transcription–Export (TREX) complex plays a crucial role in facilitating the mRNA export from the nucleus to the cytoplasm. One key function of TREX is its ability to discriminate mature mRNAs from immature precursors and other nuclear RNAs, thereby ensuring the selective export of fully processed transcripts [248]. The components of the TREX complex, in particular Alyref and Thoc5 as part of the THO subcomplex, are primarily involved in the interaction with NXF1 [249]. The depletion of both proteins has been shown to vastly decrease the NXF1 mRNA interactions but does not fully prevent complex formation. Further studies revealed that TREX induces conformational changes in NXF1 that stabilize protein–RNA interactions [250]. The recruitment of TREX is initiated during splicing via Cap Binding Complex (CBC) components as well as Exon Junction Complexes that are deposited onto the mRNA during the splicing process [251,252]. A comprehensive study of the endogenous EJCs revealed the multimerization with numerous SR proteins that, in turn, have been shown to be involved in the direct recruitment of NXF1 comparably to Alyref [253].

Thus, studies have highlighted the significance of SR proteins in their role as adaptors that link alternative RNA processing to mRNA export by connecting the splicing and 3′ end formation to the mRNA export [254]. SR proteins are generally characterized by their RRMs and an RS domain. Phosphorylation, primarily by SRPK1/2 in the cytoplasm and Clk1/4 in the nucleus, plays a pivotal role in regulating their functions. The phosphorylation by SRPKs enables the nuclear import and localization of SR proteins to nuclear speckles [255]. While the hyperphosphorylation by Clk kinases is, furthermore, crucial for their recruitment to transcription sites and subsequent spliceosome assembly, the dephosphorylation by PP1/2A phosphatases during splicing is in turn essential for releasing the splicing machinery and the recruitment of NXF1 [256], reviewed in [49]. Huang and colleagues demonstrated that the SR proteins SRSF7 and SRSF1 exhibit high affinity for NXF1 when hypo-phosphorylated, suggesting that the phosphorylation state of the SR protein adapters implies that binding occurs after splicing is completed [257]. However, since only a subset of the 12 uniformly named SR proteins are capable of nucleocytoplasmic shuttling, their interaction preference for direct NXF1 recruitment differs. While SRSF1, SRSF3, SRSF4, SRSF6, SRSF7, and SRSF10 were found to have shuttling activities and therefore can directly influence mRNA export, although with different kinetics, SRSF2, SRSF5, SRSF8, SRSF9, SRSF11, and SRSF12 are generally considered non-shuttling SR proteins [258]. The interaction between the SR proteins and NXF1 is primarily achieved via RS:RS interaction. Intriguingly, the iCLIP data from NXF1 also showed that the NXF1 protein has a binding preference for sequences that overlap with putative SRSF3 binding sites, indicating the co-binding of both proteins. In addition, a direct and strong interaction preference with the other highly kinetic shuttling SR proteins, SRSF1 and SRSF7, was demonstrated [62,254,259]. Accordingly, the downregulation of SR proteins, in particular SRSF1, SRSF3, and SRSF7, by HIV-1 infection-induced interferon responses can drastically alter the gene expression dynamics, especially for the transcripts of the 2 kb class that rely on NXF1-mediated mRNA export and thus SR protein availability. The depletion of the SR proteins in the context of HIV-1 infections has been shown to be detrimental for both virion production as well as infectivity (reviewed in [260]) primarily due to a shift in the splicing regulation but also potentially due to changes in the export regulation.

Aside from the direct regulation of nucleocytoplasmic trafficking by SR proteins, other members of the SR superfamily with SR-protein-like structures are also involved in these checkpoint processes. CPSF6 (Cleavage and Polyadenylation Specificity Factor 6), which is part of the cleavage factor I mammalian (CFIm), is crucial for the nuclear import and replication of HIV-1 in macrophages, as well as for regulating alternative polyadenylation (APA) and mRNA processing. In HIV-1 infections, it interacts with the viral capsid at the nuclear pore complex (NPC) via nucleoporins like Nup153, facilitating the translocation of the viral replication complex into the nucleus [261]. This process is primarily mediated by transportin 3 (TNPO3), which binds to the RS-like domain of CPSF6, which ultimately enables nuclear import. The phosphorylation state of the CPSF6 RS-like domain can influence its function, where hyperphosphorylated residues can disrupt its interaction with TNPO3. Furthermore, a direct interaction of SRSF1 with CPSF6 and TNPO3 has been shown to influence the coordination of the nuclear import and processing of viral RNA. The association of CPSF6 with TNPO3 and its recruitment to the viral capsid are essential for the nuclear import of HIV-1 replication complexes [262]. The disruption of CPSF6 binding through knockdown or mutations results in the accumulation of viral complexes at the nuclear envelope, which ultimately reduces the infectivity. Thus, proper CPSF6 function in APA and mRNA processing is necessary for efficient viral replication [261].

While the transcripts of the 2 kb class rely on the primary host mRNA export factor NXF1, the efficient export of the intron-containing 4 kb and unspliced 9 kb class requires the control mechanism of the NXF1-dependent mRNA export to be circumvented. Generally, intron-containing mRNAs are retained within the nucleus and marked for rapid degradation to prevent the expression of aberrant proteins [263]. Therefore, HIV-1 makes use of a feedback loop using the re-imported viral regulatory protein Rev that enables the interaction with a more specialized export factor, CRM1, also known as Exportin-1 [264]. Therefore, Rev proteins bind the roughly 350 nucleotide-long, highly structured RRE that is located in the env coding region (Figure 1) and shows a high level of conservation across different HIV-1 isolates [265]. Here, a Rev oligomer is accumulated that displays nuclear export sequences (NESs) that are recognized by the CRM1–Ran–GTP nuclear receptor complex [266]. Thus, the Rev–CRM-1 interaction and subsequent initiation of nuclear export are mediated by the Rev NES and the cargo-binding groove of CRM-1 [267]. Furthermore, interactions with the phenylalanine–glycine (FG) repeats of multiple nucleoporins contribute to the dynamics of the CRM-1-mediated nuclear export by regulating the assembly and disassembly of the export complex at the NPC [268].

Studies have shown that alterations in the subcellular distribution of Rev can significantly impact the export efficiency of RRE-containing viral mRNA species, thereby influencing the overall virus production [269]. This suggests the presence of sequences that retain transcripts within the nucleus in the absence of Rev proteins. Several analyses revealed that specific *cis*-acting repressive sequences (CRSs) within HIV-1 RNA can affect the interactions with CRM1 and other cellular factors, consequently influencing the mRNA export dynamics [270,271,272], reviewed in [3]. Intriguingly, interferon-regulated RBPs are involved in this interaction. Members of the hnRNP family have been found to be involved in the nuclear retention of viral mRNA transcripts by interaction with CRS (Figure 1) [273]. While some members of the hnRNP family exhibit rapid nucleocytoplasmic shuttling, other members, in particular hnRNP C and U, are primarily found in the nucleus [106,274]. Its strong nuclear localization signal (NLS) makes hnRNP C almost exclusively nuclear, and thus its binding to viral CRS contributes to the nuclear retention of transcripts in the absence of Rev to an extent that is even capable of overriding NES [275]. In accordance, a similar mechanism for hnRNP A2/B1 has been described. In a study by Gordon and colleagues, the expression of a Rev-negative provirus generated predominantly nuclear localized viral RNA until the hnRNP A2/B1 proteins were depleted from the cells, which resulted in the translocation of the viral RNA into the cytoplasm. This is due to hnRNP A2/B1 exhibiting targeted affinity towards particular segments (A2RE) within the HIV-1 genome that contribute to nuclear localization. Additionally, the reduced levels of hnRNP A2/B1 coincided with the heightened expression of the viral structural proteins, while the mRNA levels remained stable, which confirms the role of hnRNP A2/B1 in retaining the HIV-1 transcripts within the nucleus [276,277]. In contrast, the molecular function of hnRNP A1 in nuclear export is more complex. While it was found to synergistically interact with Rev to facilitate mRNA export as it contains both an NLS and NES, it was also described to be involved in the nuclear retention of transcripts [278,279]. The underlying mechanism that induces the switch between nuclear retention or export mediated by hnRNP A1 has not yet been described. Additionally, the M9 localization signal present in hnRNP A1 was shown to facilitate the translocation from the cytoplasm into the nucleus and vice versa [280]. Here, M9 interacts with transport receptors such as transportin-1, which mediates the transport.

The nuclear retention of unspliced HIV-1 mRNA under high expression levels was also reported for hnRNP A0. Notably, the siRNA-mediated knockdown resulted in the facilitated export of unspliced mRNA in HEK293T cells [47]. Furthermore, hnRNP A0 contains a derivate of the M9 localization signal of hnRNP A1, with the required key elements for shuttling preserved [47]. It is not unlikely that, similar to hnRNP A1 [281,282], hnRNP A0 is able to bind the *cis*-repressive elements (CREs) within the HIV-1 genome and thus inhibit the export of intron-containing mRNA [3]. However, the CRE-mediated inhibitory mechanism, and whether hnRNP A0 directly binds mRNA or modulates the export by secondary effects, still remain to be investigated.

In high-producing HIV-1-infected CD4+ T lymphocytes, Tat is localized in the nucleus, where it decreases the viral RNA splicing by diminishing the alternative splicing factors, such as hnRNP A2/B1. This results in a higher accumulation of RRE-dependent RNAs in the nucleus, which are available for Rev binding. When Rev binds to the RRE elements of viral RNA, its NES is exposed, allowing it to be exported from the nucleus to the cytoplasm with its bound viral RNA cargo. On the other hand, in low-producing astrocytes, Tat is primarily localized in the cytoplasm, with only a small fraction in the nucleus during infection. As a result, the Tat-mediated regulation of hnRNP A2/B1 is absent in astrocytes, leading to increased binding of hnRNP A2/B1 to the D1 and D4 sites, which initiates more splicing of viral mRNA. This results in lower levels of RRE-dependent RNA in the nucleus of astrocytes, preventing Rev from binding to the RRE elements. Consequently, most of the Rev remains unbound in the nucleus of astrocytes, and its NES is masked, preventing its export to the cytoplasm [283].

HIV-1 infection in macrophages per se might affect the nuclear import/export pathways used by SRSF2, such as the transporting SR system [284], or alternatively by the reversible phosphorylation of the RS domain that regulates the subcellular localization and shuttling of SR proteins [100].

Generally, HIV-1 mRNA export is characterized by a dense network of interplaying RBPs for the optimal temporal orchestration of mRNA expression. Here, interferon-regulated RBPs play a pivotal role as direct and indirect interaction partners of export proteins.

## 7. Impact of Interferon-Regulated Splicing Factors on Programmed Ribosomal Frameshifting

Many RNA viruses have polycistronic genomes and use different strategies to access downstream open reading frames (ORFs). Programmed ribosomal frameshifting (PRF), initially identified in Rous sarcoma virus in 1985 [285], is a translational mechanism in which the ribosome shifts to a different reading frame during protein synthesis that enables limited genomes to expand their genetic repertoire to express two or more proteins from a single messenger RNA. The maintenance of balanced frameshifting is often essential for preserving optimal protein ratios that are crucial for efficient viral replication. During elongation, the ribosome decodes the mRNA in triplets and maintains the reading frame. However, at the slippery site, the ribosome has the capability to backtrack by a single nucleotide (nt), leading to the utilization of the –1 ORF, thereby bypassing premature termination and facilitating the translation of a novel set of proteins [285]. The stalling of the ribosome at the slippery site is facilitated by downstream stimulatory mRNA structures such as pseudoknots [286]. Furthermore, -1PRF is widely used by several viral families, bacteria, and a small number of cellular genes. These viruses include HIV-1, human T-cell lymphotropic virus-1 (HTLV), respiratory syncytial virus (RSV), as well as endemic and highly pathogenic coronaviruses SARS-CoV, MERS-CoV, and SARS-CoV-2 [285,287,288,289,290]. In addition, the Alphavirus Chikungunya virus (CHIKV) and some flaviviruses like West Nile virus (WNV) and Japanese encephalitis virus (JEV) use -1PRF for their gene expression [286]. Cellular genes using -1PRF include the HIV-1 T-cell receptor C–C chemokine receptor 5 (CCR5) and the retrotransposon-derived gene PEG10 [285,287,288]. CCR5 uses -1PRF for premature translation termination, which then leads to destabilization and nonsense-mediated mRNA decay. In this case, -1PRF is used as a tool to regulate the protein levels during the immune response [291].

**Figure 7:**
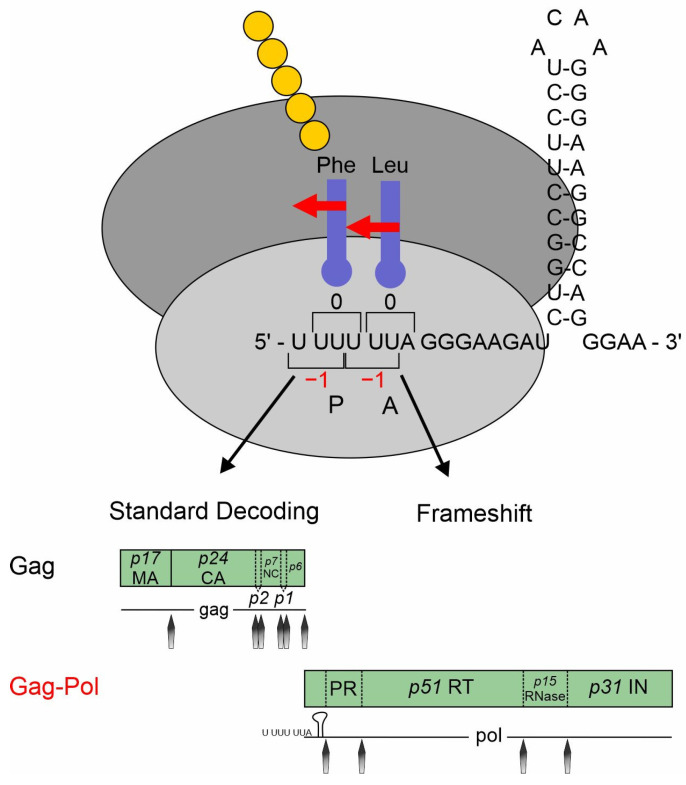
Schematic illustration of the HIV-1 programmed ribosomal frameshifting. Schematic representation depicting −1 programmed ribosomal frameshifting (-1PRF) in HIV-1. The ribosome is depicted with the slippery site highlighted, comprising the sequence “UUUUUUA”, where the frameshift occurs. Two reading frames are illustrated: *gag*–*pol* being dependent on PRF and *gag* being independent of PRF. The hairpin structure of the mRNA downstream the slippery site is discernible, aiding in understanding the mechanism of frameshifting. Red arrows indicate the frame shifting direction. Illustration was modified according to [296].

The frameshift site of retroviruses including HIV-1 is located at the overlap of the *gag* and *pol* ORFs (Figure 1). Without -1PRF, only *gag* can be translated, but, after a -1PRF event, the polyprotein Gag–Pol is expressed, enabling the translation of important proteins such as the reverse transcriptase [287]. The highly conserved slippery sequence U UUU UUA is followed by an 8 nt spacer and a single stem loop consisting of 26 nt (Figure 7) [292].

Slippery sites are optimized for the respective frameshifting event, with the downstream regions and RNA structures exerting a context-dependent influence on PRF [293]. PRF sites display varying sensitivities to alterations in the downstream sequence. Moreover, additional regulators play a role in PRF regulation. Investigations of the PRF sites within HIV-1 clinical isolates revealed subtype-specific differences and correlations with the viral load. Since the expression of Gag is sufficient for the assembly and release of viral particles, it only results in non-infectious virions lacking viral enzymes [294]. The translation of the Gag–Pol polyprotein is therefore essential for the production of infectious viral particles. The well-conserved Gag:Gag–Pol ratio for HIV-1 is 1:20 and is very sensitive to imbalance as it is affecting proteolytic processing and RNA dimerization. Even small changes are detrimental to efficient viral replication [295].

Recently, we identified hnRNP A0 as a novel host factor inhibiting HIV-1 replication by targeting different mechanisms, including -1PRF [47], demonstrating that hnRNP A0 inhibits -1PRF in a dual-luciferase reporter assay as well as during infection by analyzing the protein levels of p55 and p24 versus p51 and p15. Moreover, we demonstrated that the HIV-1 -1PRF efficiency is slightly increased during the depletion of the hnRNP A0 protein levels by siRNA-mediated knockdown. Members of the hnRNP family are also able to target -1PRF, resulting in a significant decrease by 50% in the -1PRF efficiency of SARS-CoV-2 under high hnRNP H1 and H2 levels [297].

It remains unclear whether hnRNP A0 alone mediates the frameshifting inhibition or if other cellular factors are involved. Indeed, several cellular proteins, including host restriction factors, have been described. Wang et al. discovered that the ISG Shiftless (RyDen, SFL, C19orf66) is able to efficiently inhibit HIV-1 -1PRF [298]. The authors propose that SFL binds to the target mRNA and the translating ribosome, which interferes with the frameshift process. Jäger et al. were able to show that the RRA region of SFL is responsible for binding the HIV RNA and that the multimerization of SFL is important for the interaction with the RNA and the translating ribosome [299]. SFL was described to reduce the frameshift efficiency of SARS-CoV, SARS-CoV-2, and JEV by targeting -1PRF [296,300]. SFL restricts the replication of JEV by targeting the frameshift-induced production of the non-structural protein NS1 [300]. Even though the mechanisms by which SFL affects the -1PRF of coronaviruses, including interacting with the ribosome, have been proposed, the exact mechanism is unclear. SFL has also been described to inhibit Dengue virus, Zika virus, hepatitis C virus (HCV), and Kaposi’s sarcoma herpesvirus (KSHV) by different mechanisms [225,226,227,228,229,230]. The SFL-mediated inhibition of Dengue virus replication is proposed to be due to interference with the translation of the viral RNA by inhibiting PABPC1 and LARP1 [301]. SFL limits Zika virus replication by interacting with the nonstructural protein 3 and lysosomal degradation [302]. During HCV infection, the SFL expression is upregulated and targets the formation of the membranous web, which is part of the HCV replication organelle [303]. SFL is able to restrict the expression of several early genes during KSHV infection, including ORF50 and ORF57, and is involved in the regulation of RNA stability and translation as well as RNA granule formation [304].

Another host factor limiting HIV-1 -1PRF is the ISG zinc finger antiviral protein (ZAP), which was originally discovered to inhibit murine leukemia virus (MLV) infection [305]. The short isoform of ZAP targets the -1PRF of SARS-CoV and SARS-CoV-2 by interfering with the refolding of the stimulatory pseudoknot and inhibiting viral replication [297]. In addition, ZAP is able to inhibit various viruses, including Ebola virus, Marburg virus, influenza A virus, and JEV [306,307,308,309]. The ZAP-mediated viral inhibition is mostly due to its binding to the cytosine–phosphate–guanine (CpG) dinucleotide sequences of the viral mRNA. ZAP is able to distinguish between cellular and foreign RNA since the cells show a suppressed frequency of CpG dinucleotides.

## 8. RNA-Binding Proteins and Splicing Factors Provide Novel Therapy Strategies

In contrast to antibiotics, which are effective against a wide range of bacteria, there are currently no broad-spectrum antiviral therapeutics available for the treatment of human-pathogenic viruses. While many clinically relevant RNA virus families exhibit significant genetic diversity, they often rely on similar cellular processes for replication [2]. As the viral genome becomes stably integrated into the host chromosome early in the infection process, viral reservoirs are established within the peripheral blood and lymphoid tissues. Consequently, the complete elimination of HIV-1 from infected individuals remains unfeasible using the existing antiretroviral therapy (ART) strategies [310,311]. The contemporary antiviral strategies rely on suppressing HIV-1 proteins such as integrase, reverse transcriptase, or protease. The prolonged administration of these therapies may precipitate the development of drug-resistant mutations and induce adverse reactions [312]. Of note, it has been demonstrated that the drug resistance-associated mutations within Exon 2/2b can induce alterations in HIV-1 mRNA splicing and HIV-1 gene expression [236]. However, unconventional mechanisms of viral gene expression represent promising targets for therapeutic intervention.

Given that all human cells express moderate levels of RBPs, such as SRSF1 or hnRNP A0, and harbor their transcriptional regulators in their genome, targeted activation could be used for therapeutic purposes. CRISPR-on (CRISPR activation) presents a precise and specific approach for activating the promoters and modulating the gene expression in a targeted manner. Consequently, this technology also holds significant potential for the broad-spectrum treatment of viral diseases if the viral gene expression mechanisms are comparable. Catalytically inactive Cas9, fused with a transcriptional activator peptide, can thereby specifically enhance the transcription of a particular gene. The design of the guide RNA (gRNA) sequence, which directs the dCas9 activator to the desired promoter or regulatory regions of the gene, is crucial for functionality. Of note, the CRISPR/dCas9 activator complex was also shown to be suitable as a potent and targeted activator of latent HIV-1 [313].

The potent effect of interferon-regulated RBPs SRSF1 and hnRNP A0 in inhibiting the virus replication at high levels raises the question of whether direct compound-mediated manipulation can also induce virus inhibition. Indeed, the compound IDC16, which interacts directly with the RS domain of SRSF1 and inhibits its phosphorylation, has been shown to suppress the splicing-promoting activity of SRSF1, essential for the generation of multiple spliced mRNA isoforms encoding Tat, Rev, and Nef [314,315,316]. Likewise, the compound 1C8 facilitated the dephosphorylation of SRSF10 and thereby enhanced its association with hTra2β, a protein previously implicated in the regulation of HIV-1 RNA splicing impairing HIV-1 replication, demonstrating the dephosphorylation of SR proteins as an antiviral strategy [317,318].

However, while the targeted manipulation of cellular factors may mitigate the risk of drug resistance, it can lead to significant cellular alterations and severe side effects in the long term, rendering therapeutic intervention through the broad modulation of these regulators a risky strategy. Conversely, a promising approach for chronic infections such as HIV-1 appears to be the targeted blocking of RBS, i.e., *trans*-acting proteins, by modifying their specific binding to the regulatory sequences in the viral genome. Here, the utilization of HIV-1 alternative splice sites represents a promising target for antiretroviral therapy (reviewed in [319]). Indeed, various small-molecule compounds have been identified to inhibit distinct phases of HIV-1 RNA processing, including digoxin, chlorhexidine, 8-azaguanine, or ABX464, with limited off-target effects [74,320,321,322]. Various strategies encompass gene therapy employing RNA interference, entailing the silencing of target genes via siRNAs (reviewed in [323,324]), or the utilization of an antisense U7 snRNA [325,326]. The latter is assimilated into U7 snRNP, inducing exon skipping by obscuring the splice site. Among the other RNA-based therapies for the treatment of HIV-1 infection (reviewed in [327]), the inhibition of HIV-1 replication was also demonstrated by the addition of modified U1 small nuclear RNAs, causing excessive RNA splicing [328,329]. Locked nucleic acids (LNAs) represent a promising approach with two crucial advantages over the conventional antisense oligonucleotides that are relevant for several applications in basic research, molecular diagnostic, and therapeutic strategies. LNAs exhibit high duplex stability, provide enhanced mismatch discrimination, demonstrate heightened resilience against both endo- and exonucleases, fall within a low toxicity threshold, and possess the capability to infiltrate RNA secondary structures [330,331]. Particularly, T-cells exhibit a strong capacity to internalize LNAs even in the absence of transfection reagents by an uptake process termed “gymnosis” [47,332,333,334]. However, in the transfection reagent-free approach, the RNA degradation is induced instead of masking since LNAs are not introduced into the nucleus [334]. The LNAs targeting and blocking viral *cis*-regulatory elements such as G_I3_-2, ESE_tat_, ESS2b, and ESE2b change the viral splice site use, thus mirroring the phenotype observed upon the mutation of these sequences and limiting viral replication [193,218,225]. Furthermore, LNAs were used to target various HIV-1 RNA *cis*-regulatory sequences, including TAR, the primer binding site (PBS), the dimerization initiation site (DIS), splice donor 1 (SD1), and the *gag* AUG, to block different levels of the HIV-1 replication cycle in infected T-cell lines and primary PBMCs [335,336,337,338]. LNAs can also be used to downregulate RBPs, significantly affecting the viral replication, thus rendering them a highly versatile tool [47].

Human-pathogenic RNA viruses exhibit considerable genomic diversity, resulting in a challenge in terms of the design of a broad-spectrum antiviral agent that is effective across virus families. However, RNA viruses, including highly pathogenic ones like HIV-1 and SARS-CoV-2 from the families of retroviruses and coronaviruses, respectively, share mechanistic similarities and exploit comparable cellular mechanisms to fulfill their replication cycles. Our studies have demonstrated that the cellular host factor hnRNP A0 exerts potent antiviral effects against retroviruses such as HIV-1 by inhibiting the programmed ribosomal frameshifting (-1PRF) and LTR promoter activity [47]. It is conceivable that hnRNP A0 could also impede other highly virulent human pathogens, such as coronaviruses or influenza viruses. Additionally, promoter analyses have demonstrated that SRSF1, for instance, can inhibit the promoter of JCPyV [339,340,341,342]. Human papillomavirus type 16 and adenoviruses are also influenced by SR proteins [343,344]. Considering the incorporation of retrovirus-like motifs into mammalian genes and their potential involvement in frameshifting, antiviral agents aimed at this mechanism might elicit unforeseen effects on the cellular metabolism in non-infected cells. Exploiting -1PRF as a potential target for antiviral intervention, certain compounds have been identified to disrupt the frameshift efficiency of different viruses. A synthetic compound to interfere with the -1PRF of HIV-1 (RG501) was shown to stimulate the frameshift efficiency, thus altering the Gag:Pol ratio, targeting the HIV-1 frameshifting in a highly selective manner, and limiting the infectivity [345]. Further inhibitors have been identified [346]. Also, the oligonucleotide-based targeting of the frameshift region was proven to be effective [347].

Understanding the protein structure and domain architecture of hnRNPs and SRSFs provides a molecular framework for elucidating their pivotal roles in viral RNA processing and gene expression. Deciphering the intricate interplay between hnRNPs and SRSFs and viral nucleic acid substrates holds promise for unveiling novel therapeutic targets.

## 9. Conclusions

**Figure 8:**
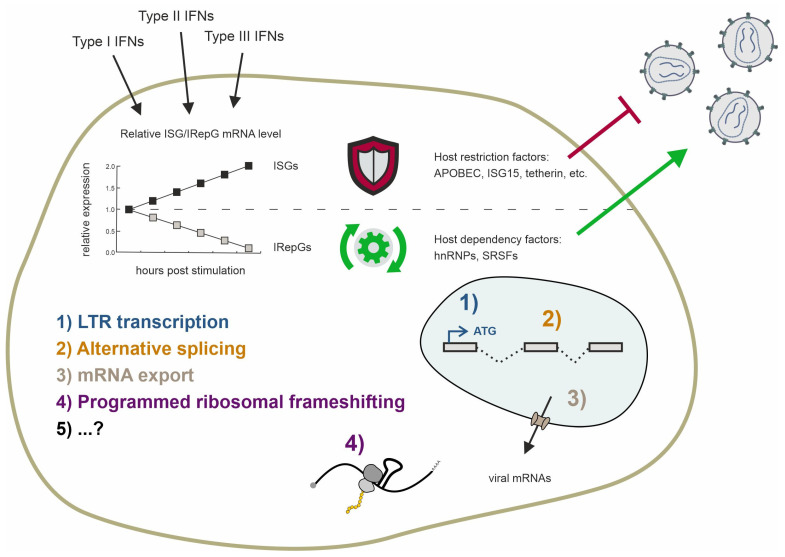
The net antiviral state of an interferon-stimulated cell is established by the interplay of host restriction and host dependency factors. Interferon (IFN) stimulation induces a cellular antiviral state, altering the expression of interferon-stimulated genes (ISGs) and interferon-repressed genes (IRepGs).

The involvement of various RNA-binding proteins in HIV-1 infection and their contribution to efficient viral replication are becoming increasingly apparent. We described a novel mechanistical model regarding how the cellular host splicing factors might also decisively impact the viral replication in the presence of interferon-induced host restriction factors. Our graphical abstract (Figure 8) illustrates the temporal expression profiles of ISGs and IRepGs in response to IFN stimulation. The ISGs, encompassing host restriction factors like APOBEC, ISG15, and tetherin, exhibit increased expression upon IFN stimulation, while the IRepGs, including cellular splicing factors such as SRSF proteins and hnRNPs, are temporarily downregulated. Despite the antiviral response, the IRepGs, notably the SRSFs and hnRNPs, facilitate viral replication by promoting (1) HIV transcription, (2) alternative splicing, (3) mRNA trafficking, and (4) programmed ribosomal frameshifting. (5) Other steps of viral replication may also be affected. Hence, the net antiviral state is determined by the balance between the ISGs and IRepGs, with the IRepGs contributing to the viral evasion and establishment of the infection during the early phase. Although individually SRSFs and hnRNPs may have only a modest impact, cumulatively, they can significantly contribute to HIV-1 replication at multiple steps despite the presence of interferon-induced host restriction factors. Future investigations will elucidate whether the initial downregulation of IRepGs represents a requisite cellular mechanism for establishing a robust antiviral milieu and whether HIV-1 is evolutionarily adapted to exploit this condition for efficient replication.

## Figures and Tables

**Table 1:** Role of interferon-regulated SRSF1 and hnRNP A0 proteins in HIV-1 gene expression.

	IFN-I-Regulated Genes			
Protein Levels	SRSF1 High	SRSF1 Low	hnRNP A0 High	hnRNP A0 Low
**transcription**	LTR inhibition, Competes with Tat-TAR-binding and trans-activation	LTR activation, increased levels of total HIV-1 mRNAs	Tat-LTR inhibition	Tat-LTR activation
**alternative splicing**	increase in *vif*, *vpr*, *tat2*, *tat3* splicing efficiency; decreased exon3, *tat1*, and US HIV-1 mRNA efficiency	increase in US HIV-1 mRNA; decreased exon2, exon3, *vif*, *vpr*, *tat2*, and MS HIV-1 mRNA splicing efficiency	increase in exon2 and *vif* mRNA splicing	no impact on alternative splice site use
**mRNA** **trafficking**	Facilitated export of unspliced 9 kb *gag*/*pol* mRNA Nuclear retention of 4 kb intron-containing *env1* mRNA	Facilitated export of 4 kb intron-containing *env1* mRNA Nuclear retainment of unspliced 9 kb *gag*/*pol* mRNA	Retains unspliced HIV-1 mRNA in the nucleus	Facilitated nuclear export of unspliced HIV-1 mRNA
**programmed ribosomal** **frameshifting**	n.d.	n.d.	Reduced -1PRF efficiency	Slightly facilitated -1PRF efficiency

MS: multiply spliced; US: unspliced; n.d.: not determined; -1PRF: -1 programmed ribosomal frameshifting; IFN-I: type I interferon.

## References

[1] Ho J.S.Y., Zhu Z., Marazzi I. (2021). Unconventional viral gene expression mechanisms as therapeutic targets. Nature.

[2] Ramdas P., Sahu A.K., Mishra T., Bhardwaj V., Chande A. (2020). From Entry to Egress: Strategic Exploitation of the Cellular Processes by HIV-1. Front. Microbiol..

[3] Ostermann P.N., Ritchie A., Ptok J., Schaal H. (2021). Let It Go: HIV-1 cis-Acting Repressive Sequences. J. Virol..

[4] Hulo C., de Castro E., Masson P., Bougueleret L., Bairoch A., Xenarios I., Le Mercier P. (2011). ViralZone: A knowledge resource to understand virus diversity. Nucleic Acids Res..

[5] Freed E.O. (2001). HIV-1 replication. Somat. Cell Mol. Genet..

[6] Rozina A., Anisenko A., Kikhai T., Silkina M., Gottikh M. (2022). Complex Relationships between HIV-1 Integrase and Its Cellular Partners. Int. J. Mol. Sci..

[7] Lusic M., Siliciano R.F. (2017). Nuclear landscape of HIV-1 infection and integration. Nat. Rev. Microbiol..

[8] Savoret J., Mesnard J.M., Gross A., Chazal N. (2020). Antisense Transcripts and Antisense Protein: A New Perspective on Human Immunodeficiency Virus Type 1. Front. Microbiol..

[9] Karn J., Stoltzfus C.M. (2012). Transcriptional and posttranscriptional regulation of HIV-1 gene expression. Cold Spring Harb. Perspect. Med..

[10] Ott M., Geyer M., Zhou Q. (2011). The control of HIV transcription: Keeping RNA polymerase II on track. Cell Host Microbe.

[11] Purcell D.F., Martin M.A. (1993). Alternative splicing of human immunodeficiency virus type 1 mRNA modulates viral protein expression, replication, and infectivity. J. Virol..

[12] Stoltzfus C.M. (2009). Chapter 1. Regulation of HIV-1 alternative RNA splicing and its role in virus replication. Adv. Virus Res..

[13] Corbeil J., Sheeter D., Genini D., Rought S., Leoni L., Du P., Ferguson M., Masys D.R., Welsh J.B., Fink J.L. (2001). Temporal gene regulation during HIV-1 infection of human CD4+ T cells. Genome Res..

[14] Wei P., Garber M.E., Fang S.M., Fischer W.H., Jones K.A. (1998). A novel CDK9-associated C-type cyclin interacts directly with HIV-1 Tat and mediates its high-affinity, loop-specific binding to TAR RNA. Cell.

[15] Shilatifard A., Conaway R.C., Conaway J.W. (2003). The RNA polymerase II elongation complex. Annu. Rev. Biochem..

[16] Kao S.Y., Calman A.F., Luciw P.A., Peterlin B.M. (1987). Anti-termination of transcription within the long terminal repeat of HIV-1 by tat gene product. Nature.

[17] Tahirov T.H., Babayeva N.D., Varzavand K., Cooper J.J., Sedore S.C., Price D.H. (2010). Crystal structure of HIV-1 Tat complexed with human P-TEFb. Nature.

[18] Demarchi F., d‘Adda di Fagagna F., Falaschi A., Giacca M. (1996). Activation of transcription factor NF-kappaB by the Tat protein of human immunodeficiency virus type 1. J. Virol..

[19] Westendorp M.O., Shatrov V.A., Schulze-Osthoff K., Frank R., Kraft M., Los M., Krammer P.H., Droge W., Lehmann V. (1995). HIV-1 Tat potentiates TNF-induced NF-kappa B activation and cytotoxicity by altering the cellular redox state. EMBO J..

[20] Pahl H.L. (1999). Activators and target genes of Rel/NF-kappaB transcription factors. Oncogene.

[21] Cochrane A., Kramer R., Ruben S., Levine J., Rosen C.A. (1989). The human immunodeficiency virus rev protein is a nuclear phosphoprotein. Virology.

[22] Malim M.H., Bohnlein S., Hauber J., Cullen B.R. (1989). Functional dissection of the HIV-1 Rev trans-activator--derivation of a trans-dominant repressor of Rev function. Cell.

[23] Fischer U., Meyer S., Teufel M., Heckel C., Luhrmann R., Rautmann G. (1994). Evidence that HIV-1 Rev directly promotes the nuclear export of unspliced RNA. EMBO J..

[24] Truman C.T., Jarvelin A., Davis I., Castello A. (2020). HIV Revisited. Open Biol..

[25] Cochrane A.W., Perkins A., Rosen C.A. (1990). Identification of sequences important in the nucleolar localization of human immunodeficiency virus Rev: Relevance of nucleolar localization to function. J. Virol..

[26] Henderson B.R., Percipalle P. (1997). Interactions between HIV Rev and nuclear import and export factors: The Rev nuclear localisation signal mediates specific binding to human importin-beta. J. Mol. Biol..

[27] Arrigo S.J., Chen I.S. (1991). Rev is necessary for translation but not cytoplasmic accumulation of HIV-1 vif, vpr, and env/vpu 2 RNAs. Genes Dev..

[28] Felber B.K., Hadzopoulou-Cladaras M., Cladaras C., Copeland T., Pavlakis G.N. (1989). rev protein of human immunodeficiency virus type 1 affects the stability and transport of the viral mRNA. Proc. Natl. Acad. Sci. USA.

[29] Gonzalez-Hernandez M.J., Cavalcoli J.D., Sartor M.A., Contreras-Galindo R., Meng F., Dai M., Dube D., Saha A.K., Gitlin S.D., Omenn G.S. (2014). Regulation of the human endogenous retrovirus K (HML-2) transcriptome by the HIV-1 Tat protein. J. Virol..

[30] Stove V., Van de Walle I., Naessens E., Coene E., Stove C., Plum J., Verhasselt B. (2005). Human immunodeficiency virus Nef induces rapid internalization of the T-cell coreceptor CD8alphabeta. J. Virol..

[31] Schwartz O., Marechal V., Le Gall S., Lemonnier F., Heard J.M. (1996). Endocytosis of major histocompatibility complex class I molecules is induced by the HIV-1 Nef protein. Nat. Med..

[32] Schindler M., Wurfl S., Benaroch P., Greenough T.C., Daniels R., Easterbrook P., Brenner M., Munch J., Kirchhoff F. (2003). Down-modulation of mature major histocompatibility complex class II and up-regulation of invariant chain cell surface expression are well-conserved functions of human and simian immunodeficiency virus nef alleles. J. Virol..

[33] Wang J.K., Kiyokawa E., Verdin E., Trono D. (2000). The Nef protein of HIV-1 associates with rafts and primes T cells for activation. Proc. Natl. Acad. Sci. USA.

[34] Raney A., Kuo L.S., Baugh L.L., Foster J.L., Garcia J.V. (2005). Reconstitution and molecular analysis of an active human immunodeficiency virus type 1 Nef/p21-activated kinase 2 complex. J. Virol..

[35] Campbell E.M., Nunez R., Hope T.J. (2004). Disruption of the actin cytoskeleton can complement the ability of Nef to enhance human immunodeficiency virus type 1 infectivity. J. Virol..

[36] Maziere J.C., Landureau J.C., Giral P., Auclair M., Fall L., Lachgar A., Achour A., Zagury D. (1994). Lovastatin inhibits HIV-1 expression in H9 human T lymphocytes cultured in cholesterol-poor medium. Biomed. Pharmacother..

[37] Harris R.S., Bishop K.N., Sheehy A.M., Craig H.M., Petersen-Mahrt S.K., Watt I.N., Neuberger M.S., Malim M.H. (2003). DNA deamination mediates innate immunity to retroviral infection. Cell.

[38] Sheehy A.M., Gaddis N.C., Choi J.D., Malim M.H. (2002). Isolation of a human gene that inhibits HIV-1 infection and is suppressed by the viral Vif protein. Nature.

[39] Sheehy A.M., Gaddis N.C., Malim M.H. (2003). The antiretroviral enzyme APOBEC3G is degraded by the proteasome in response to HIV-1 Vif. Nat. Med..

[40] Lubow J., Collins K.L. (2020). Vpr Is a VIP: HIV Vpr and Infected Macrophages Promote Viral Pathogenesis. Viruses.

[41] Vanegas-Torres C.A., Schindler M. (2024). HIV-1 Vpr Functions in Primary CD4(+) T Cells. Viruses.

[42] West C.M. (2003). Evolutionary and functional implications of the complex glycosylation of Skp1, a cytoplasmic/nuclear glycoprotein associated with polyubiquitination. Cell. Mol. Life Sci..

[43] Sertznig H., Hillebrand F., Erkelenz S., Schaal H., Widera M. (2018). Behind the scenes of HIV-1 replication: Alternative splicing as the dependency factor on the quiet. Virology.

[44] Ocwieja K.E., Sherrill-Mix S., Mukherjee R., Custers-Allen R., David P., Brown M., Wang S., Link D.R., Olson J., Travers K. (2012). Dynamic regulation of HIV-1 mRNA populations analyzed by single-molecule enrichment and long-read sequencing. Nucleic Acids Res..

[45] Schwartz S., Felber B.K., Benko D.M., Fenyo E.M., Pavlakis G.N. (1990). Cloning and functional analysis of multiply spliced mRNA species of human immunodeficiency virus type 1. J. Virol..

[46] Sertznig H., Roesmann F., Wilhelm A., Heininger D., Bleekmann B., Elsner C., Santiago M., Schuhenn J., Karakoese Z., Benatzy Y. (2022). SRSF1 acts as an IFN-I-regulated cellular dependency factor decisively affecting HIV-1 post-integration steps. Front. Immunol..

[47] Roesmann F., Sertznig H., Klaassen K., Wilhelm A., Heininger D., Elsner C., Marschalek R., Santiago M., Esser S., Sutter K. (2024). The interferon regulated host factor hnRNP A0 modulates HIV-1 replication by interference with LTR-activity, mRNA trafficking, and programmed ribosomal frameshifting. J. Virol..

[48] Fu X.D. (1995). The superfamily of arginine/serine-rich splicing factors. RNA.

[49] Howard J.M., Sanford J.R. (2015). The RNAissance family: SR proteins as multifaceted regulators of gene expression. Wiley Interdiscip. Rev. RNA.

[50] Manley J.L., Krainer A.R. (2010). A rational nomenclature for serine/arginine-rich protein splicing factors (SR proteins). Genes Dev..

[51] Shepard P.J., Hertel K.J. (2009). The SR protein family. Genome Biol..

[52] Uhlen M., Fagerberg L., Hallstrom B.M., Lindskog C., Oksvold P., Mardinoglu A., Sivertsson A., Kampf C., Sjostedt E., Asplund A. (2015). Proteomics. Tissue-based map of the human proteome. Science.

[53] Anko M.L. (2014). Regulation of gene expression programmes by serine-arginine rich splicing factors. Semin. Cell Dev. Biol..

[54] Kannan N., Neuwald A.F. (2004). Evolutionary constraints associated with functional specificity of the CMGC protein kinases MAPK, CDK, GSK, SRPK, DYRK, and CK2alpha. Protein Sci. A Publ. Protein Soc..

[55] Liu Q., Dreyfuss G. (1995). In vivo and in vitro arginine methylation of RNA-binding proteins. Mol. Cell. Biol..

[56] Siebel C.W., Guthrie C. (1996). The essential yeast RNA binding protein Np13p is methylated. Proc. Natl. Acad. Sci. USA.

[57] Yun C.Y., Fu X.D. (2000). Conserved SR protein kinase functions in nuclear import and its action is counteracted by arginine methylation in Saccharomyces cerevisiae. J. Cell Biol..

[58] Choudhary C., Kumar C., Gnad F., Nielsen M.L., Rehman M., Walther T.C., Olsen J.V., Mann M. (2009). Lysine acetylation targets protein complexes and co-regulates major cellular functions. Science.

[59] Loomis R.J., Naoe Y., Parker J.B., Savic V., Bozovsky M.R., Macfarlan T., Manley J.L., Chakravarti D. (2009). Chromatin binding of SRp20 and ASF/SF2 and dissociation from mitotic chromosomes is modulated by histone H3 serine 10 phosphorylation. Mol. Cell.

[60] Ji X., Zhou Y., Pandit S., Huang J., Li H., Lin C.Y., Xiao R., Burge C.B., Fu X.D. (2013). SR proteins collaborate with 7SK and promoter-associated nascent RNA to release paused polymerase. Cell.

[61] Huang Y., Steitz J.A. (2001). Splicing factors SRp20 and 9G8 promote the nucleocytoplasmic export of mRNA. Mol. Cell.

[62] Huang Y., Gattoni R., Stevenin J., Steitz J.A. (2003). SR splicing factors serve as adapter proteins for TAP-dependent mRNA export. Mol. Cell.

[63] Huang Y., Steitz J.A. (2005). SRprises along a messenger’s journey. Mol. Cell.

[64] Li X., Manley J.L. (2005). Inactivation of the SR protein splicing factor ASF/SF2 results in genomic instability. Cell.

[65] Xiao S.H., Manley J.L. (1997). Phosphorylation of the ASF/SF2 RS domain affects both protein-protein and protein-RNA interactions and is necessary for splicing. Genes Dev..

[66] Lai M.C., Lin R.I., Tarn W.Y. (2003). Differential effects of hyperphosphorylation on splicing factor SRp55. Biochem. J..

[67] Colwill K., Pawson T., Andrews B., Prasad J., Manley J.L., Bell J.C., Duncan P.I. (1996). The Clk/Sty protein kinase phosphorylates SR splicing factors and regulates their intranuclear distribution. EMBO J..

[68] Gui J.F., Lane W.S., Fu X.D. (1994). A serine kinase regulates intracellular localization of splicing factors in the cell cycle. Nature.

[69] Zhou Z., Fu X.D. (2013). Regulation of splicing by SR proteins and SR protein-specific kinases. Chromosoma.

[70] Czubaty A., Piekielko-Witkowska A. (2017). Protein kinases that phosphorylate splicing factors: Roles in cancer development, progression and possible therapeutic options. Int. J. Biochem. Cell Biol..

[71] Duncan P.I., Stojdl D.F., Marius R.M., Bell J.C. (1997). In vivo regulation of alternative pre-mRNA splicing by the Clk1 protein kinase. Mol. Cell. Biol..

[72] Prasad J., Colwill K., Pawson T., Manley J.L. (1999). The protein kinase Clk/Sty directly modulates SR protein activity: Both hyper- and hypophosphorylation inhibit splicing. Mol. Cell. Biol..

[73] Dahal S., Clayton K., Cabral T., Cheng R., Jahanshahi S., Ahmed C., Koirala A., Villasmil Ocando A., Malty R., Been T. (2023). On a path toward a broad-spectrum anti-viral: Inhibition of HIV-1 and coronavirus replication by SR kinase inhibitor harmine. J. Virol..

[74] Wong R., Balachandran A., Mao A.Y., Dobson W., Gray-Owen S., Cochrane A. (2011). Differential effect of CLK SR Kinases on HIV-1 gene expression: Potential novel targets for therapy. Retrovirology.

[75] Ge H., Manley J.L. (1990). A protein factor, ASF, controls cell-specific alternative splicing of SV40 early pre-mRNA in vitro. Cell.

[76] Krainer A.R., Conway G.C., Kozak D. (1990). The essential pre-mRNA splicing factor SF2 influences 5′ splice site selection by activating proximal sites. Cell.

[77] Liu X., Kraus W.L., Bai X. (2015). Ready, pause, go: Regulation of RNA polymerase II pausing and release by cellular signaling pathways. Trends Biochem. Sci..

[78] Caputi M., Freund M., Kammler S., Asang C., Schaal H. (2004). A bidirectional SF2/ASF- and SRp40-dependent splicing enhancer regulates human immunodeficiency virus type 1 rev, env, vpu, and nef gene expression. J. Virol..

[79] Kammler S., Otte M., Hauber I., Kjems J., Hauber J., Schaal H. (2006). The strength of the HIV-1 3′ splice sites affects Rev function. Retrovirology.

[80] Zhou H., Bulek K., Li X., Herjan T., Yu M., Qian W., Wang H., Zhou G., Chen X., Yang H. (2017). IRAK2 directs stimulus-dependent nuclear export of inflammatory mRNAs. Elife.

[81] Michlewski G., Sanford J.R., Cáceres J.F. (2008). The splicing factor SF2/ASF regulates translation initiation by enhancing phosphorylation of 4E-BP1. Mol. Cell.

[82] Sanford J.R., Gray N.K., Beckmann K., Caceres J.F. (2004). A novel role for shuttling SR proteins in mRNA translation. Genes Dev..

[83] Aznarez I., Nomakuchi T.T., Tetenbaum-Novatt J., Rahman M.A., Fregoso O., Rees H., Krainer A.R. (2018). Mechanism of Nonsense-Mediated mRNA Decay Stimulation by Splicing Factor SRSF1. Cell Rep..

[84] Sun S., Zhang Z., Sinha R., Karni R., Krainer A.R. (2010). SF2/ASF autoregulation involves multiple layers of post-transcriptional and translational control. Nat. Struct. Mol. Biol..

[85] Zhang Z., Krainer A.R. (2004). Involvement of SR proteins in mRNA surveillance. Mol. Cell.

[86] Tacke R., Manley J.L. (1995). The human splicing factors ASF/SF2 and SC35 possess distinct, functionally significant RNA binding specificities. EMBO J..

[87] Sanford J.R., Wang X., Mort M., Vanduyn N., Cooper D.N., Mooney S.D., Edenberg H.J., Liu Y. (2009). Splicing factor SFRS1 recognizes a functionally diverse landscape of RNA transcripts. Genome Res..

[88] Wang J., Smith P.J., Krainer A.R., Zhang M.Q. (2005). Distribution of SR protein exonic splicing enhancer motifs in human protein-coding genes. Nucleic Acids Res..

[89] Jeong S. (2017). SR Proteins: Binders, Regulators, and Connectors of RNA. Mol. Cells.

[90] Jiang L., Huang J., Higgs B.W., Hu Z., Xiao Z., Yao X., Conley S., Zhong H., Liu Z., Brohawn P. (2016). Genomic Landscape Survey Identifies SRSF1 as a Key Oncodriver in Small Cell Lung Cancer. PLoS Genet..

[91] Karni R., de Stanchina E., Lowe S.W., Sinha R., Mu D., Krainer A.R. (2007). The gene encoding the splicing factor SF2/ASF is a proto-oncogene. Nat. Struct. Mol. Biol..

[92] Das S., Krainer A.R. (2014). Emerging functions of SRSF1, splicing factor and oncoprotein, in RNA metabolism and cancer. Mol. Cancer Res..

[93] Staffa A., Cochrane A. (1995). Identification of positive and negative splicing regulatory elements within the terminal tat-rev exon of human immunodeficiency virus type 1. Mol. Cell. Biol..

[94] Jablonski J.A., Caputi M. (2009). Role of cellular RNA processing factors in human immunodeficiency virus type 1 mRNA metabolism, replication, and infectivity. J. Virol..

[95] Jacquenet S., Decimo D., Muriaux D., Darlix J.L. (2005). Dual effect of the SR proteins ASF/SF2, SC35 and 9G8 on HIV-1 RNA splicing and virion production. Retrovirology.

[96] Ropers D., Ayadi L., Gattoni R., Jacquenet S., Damier L., Branlant C., Stevenin J. (2004). Differential effects of the SR proteins 9G8, SC35, ASF/SF2, and SRp40 on the utilization of the A1 to A5 splicing sites of HIV-1 RNA. J. Biol. Chem..

[97] Koizumi J., Okamoto Y., Onogi H., Mayeda A., Krainer A.R., Hagiwara M. (1999). The subcellular localization of SF2/ASF is regulated by direct interaction with SR protein kinases (SRPKs). J. Biol. Chem..

[98] Misteli T., Spector D.L. (1996). Serine/threonine phosphatase 1 modulates the subnuclear distribution of pre-mRNA splicing factors. Mol. Biol. Cell.

[99] Novoyatleva T., Heinrich B., Tang Y., Benderska N., Butchbach M.E., Lorson C.L., Lorson M.A., Ben-Dov C., Fehlbaum P., Bracco L. (2008). Protein phosphatase 1 binds to the RNA recognition motif of several splicing factors and regulates alternative pre-mRNA processing. Hum. Mol. Genet..

[100] Sanford J.R., Ellis J.D., Cazalla D., Caceres J.F. (2005). Reversible phosphorylation differentially affects nuclear and cytoplasmic functions of splicing factor 2/alternative splicing factor. Proc. Natl. Acad. Sci. USA.

[101] Sinha R., Allemand E., Zhang Z., Karni R., Myers M.P., Krainer A.R. (2010). Arginine methylation controls the subcellular localization and functions of the oncoprotein splicing factor SF2/ASF. Mol. Cell. Biol..

[102] Paz S., Ritchie A., Mauer C., Caputi M. (2021). The RNA binding protein SRSF1 is a master switch of gene expression and regulation in the immune system. Cytokine Growth Factor Rev..

[103] Mo S., Ji X., Fu X.D. (2013). Unique role of SRSF2 in transcription activation and diverse functions of the SR and hnRNP proteins in gene expression regulation. Transcription.

[104] Yang J.F., You J. (2020). Regulation of Polyomavirus Transcription by Viral and Cellular Factors. Viruses.

[105] Dreyfuss G., Matunis M.J., Piñol-Roma S., Burd C.G. (1993). hnRNP proteins and the biogenesis of mRNA. Annu. Rev. Biochem..

[106] Geuens T., Bouhy D., Timmerman V. (2016). The hnRNP family: Insights into their role in health and disease. Hum. Genet..

[107] Hamilton B.J., Burns C.M., Nichols R.C., Rigby W.F. (1997). Modulation of AUUUA response element binding by heterogeneous nuclear ribonucleoprotein A1 in human T lymphocytes. The roles of cytoplasmic location, transcription, and phosphorylation. J. Biol. Chem..

[108] Görlach M., Wittekind M., Beckman R.A., Mueller L., Dreyfuss G. (1992). Interaction of the RNA-binding domain of the hnRNP C proteins with RNA. EMBO J..

[109] Dominguez C., Fisette J.F., Chabot B., Allain F.H. (2010). Structural basis of G-tract recognition and encaging by hnRNP F quasi-RRMs. Nat. Struct. Mol. Biol..

[110] Samatanga B., Dominguez C., Jelesarov I., Allain F.H. (2013). The high kinetic stability of a G-quadruplex limits hnRNP F qRRM3 binding to G-tract RNA. Nucleic Acids Res..

[111] Burd C.G., Dreyfuss G. (1994). Conserved structures and diversity of functions of RNA-binding proteins. Science.

[112] Kirking D.M., Thomas J.W., Ascione F.J., Boyd E.L. (1986). Detecting and preventing adverse drug interactions: The potential contribution of computers in pharmacies. Soc. Sci. Med..

[113] Marenda M., Lazarova E., Gilbert N. (2022). The role of SAF-A/hnRNP U in regulating chromatin structure. Curr. Opin. Genet. Dev..

[114] Shishkin S.S., Kovalev L.I., Pashintseva N.V., Kovaleva M.A., Lisitskaya K. (2019). Heterogeneous Nuclear Ribonucleoproteins Involved in the Functioning of Telomeres in Malignant Cells. Int. J. Mol. Sci..

[115] Ford L.P., Wright W.E., Shay J.W. (2002). A model for heterogeneous nuclear ribonucleoproteins in telomere and telomerase regulation. Oncogene.

[116] Liu Y., Abula A., Xiao H., Guo H., Li T., Zheng L., Chen B., Nguyen H.C., Ji X. (2023). Structural Insight Into hnRNP A2/B1 Homodimerization and DNA Recognition. J. Mol. Biol..

[117] Cartegni L., Maconi M., Morandi E., Cobianchi F., Riva S., Biamonti G. (1996). hnRNP A1 selectively interacts through its Gly-rich domain with different RNA-binding proteins. J. Mol. Biol..

[118] Biamonti G., Riva S. (1994). New insights into the auxiliary domains of eukaryotic RNA binding proteins. FEBS Lett..

[119] Dreyfuss G., Kim V.N., Kataoka N. (2002). Messenger-RNA-binding proteins and the messages they carry. Nat. Rev. Mol. Cell Biol..

[120] Busch A., Hertel K.J. (2012). Evolution of SR protein and hnRNP splicing regulatory factors. Wiley Interdiscip. Rev. RNA.

[121] Black D.L. (2003). Mechanisms of alternative pre-messenger RNA splicing. Annu. Rev. Biochem..

[122] Martinez-Contreras R., Cloutier P., Shkreta L., Fisette J.F., Revil T., Chabot B. (2007). hnRNP proteins and splicing control. Adv. Exp. Med. Biol..

[123] Kutluay S.B., Emery A., Penumutchu S.R., Townsend D., Tenneti K., Madison M.K., Stukenbroeker A.M., Powell C., Jannain D., Tolbert B.S. (2019). Genome-Wide Analysis of Heterogeneous Nuclear Ribonucleoprotein (hnRNP) Binding to HIV-1 RNA Reveals a Key Role for hnRNP H1 in Alternative Viral mRNA Splicing. J. Virol..

[124] Kim J.H., Hahm B., Kim Y.K., Choi M., Jang S.K. (2000). Protein-protein interaction among hnRNPs shuttling between nucleus and cytoplasm. J. Mol. Biol..

[125] Zhang X., Flavell R.A., Li H.B. (2019). hnRNPA2B1: A nuclear DNA sensor in antiviral immunity. Cell Res..

[126] Hallay H., Locker N., Ayadi L., Ropers D., Guittet E., Branlant C. (2006). Biochemical and NMR study on the competition between proteins SC35, SRp40, and heterogeneous nuclear ribonucleoprotein A1 at the HIV-1 Tat exon 2 splicing site. J. Biol. Chem..

[127] Krecic A.M., Swanson M.S. (1999). hnRNP complexes: Composition, structure, and function. Curr. Opin. Cell Biol..

[128] Fuentes Y., Olguín V., López-Ulloa B., Mendonça D., Ramos H., Abdalla A.L., Guajardo-Contreras G., Niu M., Rojas-Araya B., Mouland A.J. (2024). Heterogeneous nuclear ribonucleoprotein K promotes cap-independent translation initiation of retroviral mRNAs. Nucleic Acids Res..

[129] Piñol-Roma S., Dreyfuss G. (1992). Shuttling of pre-mRNA binding proteins between nucleus and cytoplasm. Nature.

[130] England W.E., Wang J., Chen S., Baldi P., Flynn R.A., Spitale R.C. (2022). An atlas of posttranslational modifications on RNA binding proteins. Nucleic Acids Res..

[131] Thibault P.A., Ganesan A., Kalyaanamoorthy S., Clarke J.W.E., Salapa H.E., Levin M.C. (2021). hnRNP A/B Proteins: An Encyclopedic Assessment of Their Roles in Homeostasis and Disease. Biology.

[132] Jean-Philippe J., Paz S., Caputi M. (2013). hnRNP A1: The Swiss army knife of gene expression. Int. J. Mol. Sci..

[133] Rousseau S., Morrice N., Peggie M., Campbell D.G., Gaestel M., Cohen P. (2002). Inhibition of SAPK2a/p38 prevents hnRNP A0 phosphorylation by MAPKAP-K2 and its interaction with cytokine mRNAs. EMBO J..

[134] Cannell I.G., Merrick K.A., Morandell S., Zhu C.Q., Braun C.J., Grant R.A., Cameron E.R., Tsao M.S., Hemann M.T., Yaffe M.B. (2015). A Pleiotropic RNA-Binding Protein Controls Distinct Cell Cycle Checkpoints to Drive Resistance of p53-Defective Tumors to Chemotherapy. Cancer Cell.

[135] Konishi H., Fujiya M., Kashima S., Sakatani A., Dokoshi T., Ando K., Ueno N., Iwama T., Moriichi K., Tanaka H. (2020). A tumor-specific modulation of heterogeneous ribonucleoprotein A0 promotes excessive mitosis and growth in colorectal cancer cells. Cell Death Dis..

[136] Chen C.Y., Shyu A.B. (1995). AU-rich elements: Characterization and importance in mRNA degradation. Trends Biochem. Sci..

[137] Myer V.E., Steitz J.A. (1995). Isolation and characterization of a novel, low abundance hnRNP protein: A0. RNA.

[138] Young D.J., Stoddart A., Nakitandwe J., Chen S.C., Qian Z., Downing J.R., Le Beau M.M. (2014). Knockdown of Hnrnpa0, a del(5q) gene, alters myeloid cell fate in murine cells through regulation of AU-rich transcripts. Haematologica.

[139] Schneider R., Campbell M., Nasioulas G., Felber B.K., Pavlakis G.N. (1997). Inactivation of the human immunodeficiency virus type 1 inhibitory elements allows Rev-independent expression of Gag and Gag/protease and particle formation. J. Virol..

[140] de Veer M.J., Holko M., Frevel M., Walker E., Der S., Paranjape J.M., Silverman R.H., Williams B.R. (2001). Functional classification of interferon-stimulated genes identified using microarrays. J. Leukoc. Biol..

[141] Billiau A., Matthys P. (2009). Interferon-gamma: A historical perspective. Cytokine Growth Factor Rev..

[142] McKenzie A.N.J., Spits H., Eberl G. (2014). Innate lymphoid cells in inflammation and immunity. Immunity.

[143] Kang S., Brown H.M., Hwang S. (2018). Direct Antiviral Mechanisms of Interferon-Gamma. Immune Netw..

[144] Weissmann C., Weber H. (1986). The interferon genes. Prog. Nucleic Acid Res. Mol. Biol..

[145] Hardy M.P., Owczarek C.M., Jermiin L.S., Ejdeback M., Hertzog P.J. (2004). Characterization of the type I interferon locus and identification of novel genes. Genomics.

[146] Guo K., Shen G., Kibbie J., Gonzalez T., Dillon S.M., Smith H.A., Cooper E.H., Lavender K., Hasenkrug K.J., Sutter K. (2020). Qualitative Differences Between the IFNalpha subtypes and IFNbeta Influence Chronic Mucosal HIV-1 Pathogenesis. PLoS Pathog..

[147] Karakoese Z., Ingola M., Sitek B., Dittmer U., Sutter K. (2024). IFNalpha Subtypes in HIV Infection and Immunity. Viruses.

[148] Schneider W.M., Chevillotte M.D., Rice C.M. (2014). Interferon-stimulated genes: A complex web of host defenses. Annu. Rev. Immunol..

[149] Gad H.H., Hamming O.J., Hartmann R. (2010). The structure of human interferon lambda and what it has taught us. J. Interferon Cytokine Res. Off. J. Int. Soc. Interferon Cytokine Res..

[150] Kotenko S.V., Gallagher G., Baurin V.V., Lewis-Antes A., Shen M., Shah N.K., Langer J.A., Sheikh F., Dickensheets H., Donnelly R.P. (2003). IFN-lambdas mediate antiviral protection through a distinct class II cytokine receptor complex. Nat. Immunol..

[151] Sheppard P., Kindsvogel W., Xu W., Henderson K., Schlutsmeyer S., Whitmore T.E., Kuestner R., Garrigues U., Birks C., Roraback J. (2003). IL-28, IL-29 and their class II cytokine receptor IL-28R. Nat. Immunol..

[152] Lazear H.M., Nice T.J., Diamond M.S. (2015). Interferon-lambda: Immune Functions at Barrier Surfaces and Beyond. Immunity.

[153] Josephson K., Logsdon N.J., Walter M.R. (2001). Crystal structure of the IL-10/IL-10R1 complex reveals a shared receptor binding site. Immunity.

[154] Logsdon N.J., Jones B.C., Allman J.C., Izotova L., Schwartz B., Pestka S., Walter M.R. (2004). The IL-10R2 binding hot spot on IL-22 is located on the N-terminal helix and is dependent on N-linked glycosylation. J. Mol. Biol..

[155] Gad H.H., Dellgren C., Hamming O.J., Vends S., Paludan S.R., Hartmann R. (2009). Interferon-lambda is functionally an interferon but structurally related to the interleukin-10 family. J. Biol. Chem..

[156] Mesev E.V., LeDesma R.A., Ploss A. (2019). Decoding type I and III interferon signalling during viral infection. Nat. Microbiol..

[157] Lavender K.J., Gibbert K., Peterson K.E., Van Dis E., Francois S., Woods T., Messer R.J., Gawanbacht A., Muller J.A., Munch J. (2016). Interferon Alpha Subtype-Specific Suppression of HIV-1 Infection In Vivo. J. Virol..

[158] Harper M.S., Guo K., Gibbert K., Lee E.J., Dillon S.M., Barrett B.S., McCarter M.D., Hasenkrug K.J., Dittmer U., Wilson C.C. (2015). Interferon-alpha Subtypes in an Ex Vivo Model of Acute HIV-1 Infection: Expression, Potency and Effector Mechanisms. PLoS Pathog..

[159] Li Y., Sun B., Esser S., Jessen H., Streeck H., Widera M., Yang R., Dittmer U., Sutter K. (2017). Expression Pattern of Individual IFNA Subtypes in Chronic HIV Infection. J. Interferon Cytokine Res. Off. J. Int. Soc. Interferon Cytokine Res..

[160] Moll H.P., Maier T., Zommer A., Lavoie T., Brostjan C. (2011). The differential activity of interferon-alpha subtypes is consistent among distinct target genes and cell types. Cytokine.

[161] Dolken L., Ruzsics Z., Radle B., Friedel C.C., Zimmer R., Mages J., Hoffmann R., Dickinson P., Forster T., Ghazal P. (2008). High-resolution gene expression profiling for simultaneous kinetic parameter analysis of RNA synthesis and decay. RNA.

[162] Trilling M., Bellora N., Rutkowski A.J., de Graaf M., Dickinson P., Robertson K., Prazeres da Costa O., Ghazal P., Friedel C.C., Alba M.M. (2013). Deciphering the modulation of gene expression by type I and II interferons combining 4sU-tagging, translational arrest and in silico promoter analysis. Nucleic Acids Res..

[163] Brass A.L., Dykxhoorn D.M., Benita Y., Yan N., Engelman A., Xavier R.J., Lieberman J., Elledge S.J. (2008). Identification of host proteins required for HIV infection through a functional genomic screen. Science.

[164] Konig R., Zhou Y., Elleder D., Diamond T.L., Bonamy G.M., Irelan J.T., Chiang C.Y., Tu B.P., De Jesus P.D., Lilley C.E. (2008). Global analysis of host-pathogen interactions that regulate early-stage HIV-1 replication. Cell.

[165] Zhou H., Xu M., Huang Q., Gates A.T., Zhang X.D., Castle J.C., Stec E., Ferrer M., Strulovici B., Hazuda D.J. (2008). Genome-scale RNAi screen for host factors required for HIV replication. Cell Host Microbe.

[166] Megger D.A., Philipp J., Le-Trilling V.T.K., Sitek B., Trilling M. (2017). Deciphering of the Human Interferon-Regulated Proteome by Mass Spectrometry-Based Quantitative Analysis Reveals Extent and Dynamics of Protein Induction and Repression. Front. Immunol..

[167] Lehmann C., Taubert D., Jung N., Fatkenheuer G., van Lunzen J., Hartmann P., Romerio F. (2009). Preferential upregulation of interferon-alpha subtype 2 expression in HIV-1 patients. AIDS Res. Hum. Retroviruses.

[168] Butic A.B., Spencer S.A., Shaheen S.K., Lukacher A.E. (2023). Polyomavirus Wakes Up and Chooses Neurovirulence. Viruses.

[169] Rocchi A., Sariyer I.K., Berger J.R. (2023). Revisiting JC virus and progressive multifocal leukoencephalopathy. J. Neurovirology.

[170] Guo L., Liu J.J., Long S.Y., Wang P.Y., Li S., Wang J.L., Wei X.F., Li J., Lei L., Huang A.L. (2024). TIM22 and TIM29 inhibit HBV replication by up-regulating SRSF1 expression. J. Med. Virol..

[171] Xiong Z., Shaibani A., Li Y.P., Yan Y., Zhang S., Yang Y., Yang F., Wang H., Yang X.F. (2006). Alternative splicing factor ASF/SF2 is down regulated in inflamed muscle. J. Clin. Pathol..

[172] Kontoyiannis D., Pasparakis M., Pizarro T.T., Cominelli F., Kollias G. (1999). Impaired on/off regulation of TNF biosynthesis in mice lacking TNF AU-rich elements: Implications for joint and gut-associated immunopathologies. Immunity.

[173] The ENCODE Project Consortium (2012). An integrated encyclopedia of DNA elements in the human genome. Nature.

[174] Luo Y., Hitz B.C., Gabdank I., Hilton J.A., Kagda M.S., Lam B., Myers Z., Sud P., Jou J., Lin K. (2020). New developments on the Encyclopedia of DNA Elements (ENCODE) data portal. Nucleic Acids Res..

[175] Lopez-Huertas M.R., Palladino C., Garrido-Arquero M., Esteban-Cartelle B., Sanchez-Carrillo M., Martinez-Roman P., Martin-Carbonero L., Ryan P., Dominguez-Dominguez L., Santos I.L. (2019). HCV-coinfection is related to an increased HIV-1 reservoir size in cART-treated HIV patients: A cross-sectional study. Sci. Rep..

[176] Martinez-Roman P., Lopez-Huertas M.R., Crespo-Bermejo C., Arca-Lafuente S., Cortegano I., Valle-Millares D., Gaspar M.L., Martin-Carbonero L., Dominguez-Dominguez L., Ryan P. (2020). Hepatitis C Virus Influences HIV-1 Viral Splicing in Coinfected Patients. J. Clin. Med..

[177] Jarboui M.A., Bidoia C., Woods E., Roe B., Wynne K., Elia G., Hall W.W., Gautier V.W. (2012). Nucleolar protein trafficking in response to HIV-1 Tat: Rewiring the nucleolus. PLoS ONE.

[178] Dowling D., Nasr-Esfahani S., Tan C.H., O‘Brien K., Howard J.L., Jans D.A., Purcell D.F., Stoltzfus C.M., Sonza S. (2008). HIV-1 infection induces changes in expression of cellular splicing factors that regulate alternative viral splicing and virus production in macrophages. Retrovirology.

[179] Maldarelli F., Xiang C., Chamoun G., Zeichner S.L. (1998). The expression of the essential nuclear splicing factor SC35 is altered by human immunodeficiency virus infection. Virus Res..

[180] Paz S., Krainer A.R., Caputi M. (2014). HIV-1 transcription is regulated by splicing factor SRSF1. Nucleic Acids Res..

[181] D‘Orso I., Frankel A.D. (2010). RNA-mediated displacement of an inhibitory snRNP complex activates transcription elongation. Nat. Struct. Mol. Biol..

[182] Barboric M., Yik J.H., Czudnochowski N., Yang Z., Chen R., Contreras X., Geyer M., Matija Peterlin B., Zhou Q. (2007). Tat competes with HEXIM1 to increase the active pool of P-TEFb for HIV-1 transcription. Nucleic Acids Res..

[183] Sedore S.C., Byers S.A., Biglione S., Price J.P., Maury W.J., Price D.H. (2007). Manipulation of P-TEFb control machinery by HIV: Recruitment of P-TEFb from the large form by Tat and binding of HEXIM1 to TAR. Nucleic Acids Res..

[184] Muniz L., Egloff S., Ughy B., Jady B.E., Kiss T. (2010). Controlling cellular P-TEFb activity by the HIV-1 transcriptional transactivator Tat. PLoS Pathog..

[185] Sobhian B., Laguette N., Yatim A., Nakamura M., Levy Y., Kiernan R., Benkirane M. (2010). HIV-1 Tat assembles a multifunctional transcription elongation complex and stably associates with the 7SK snRNP. Mol. Cell.

[186] AJ C.Q., Bugai A., Barboric M. (2016). Cracking the control of RNA polymerase II elongation by 7SK snRNP and P-TEFb. Nucleic Acids Res..

[187] Paz S., Caputi M. (2015). SRSF1 inhibition of HIV-1 gene expression. Oncotarget.

[188] Krueger B.J., Jeronimo C., Roy B.B., Bouchard A., Barrandon C., Byers S.A., Searcey C.E., Cooper J.J., Bensaude O., Cohen E.A. (2008). LARP7 is a stable component of the 7SK snRNP while P-TEFb, HEXIM1 and hnRNP A1 are reversibly associated. Nucleic Acids Res..

[189] Van Herreweghe E., Egloff S., Goiffon I., Jady B.E., Froment C., Monsarrat B., Kiss T. (2007). Dynamic remodelling of human 7SK snRNP controls the nuclear level of active P-TEFb. EMBO J..

[190] Burd C.G., Dreyfuss G. (1994). RNA binding specificity of hnRNP A1: Significance of hnRNP A1 high-affinity binding sites in pre-mRNA splicing. EMBO J..

[191] Jeang K.T., Xiao H., Rich E.A. (1999). Multifaceted activities of the HIV-1 transactivator of transcription, Tat. J. Biol. Chem..

[192] Li L., Dahiya S., Kortagere S., Aiamkitsumrit B., Cunningham D., Pirrone V., Nonnemacher M.R., Wigdahl B. (2012). Impact of Tat Genetic Variation on HIV-1 Disease. Adv. Virol..

[193] Erkelenz S., Hillebrand F., Widera M., Theiss S., Fayyaz A., Degrandi D., Pfeffer K., Schaal H. (2015). Balanced splicing at the Tat-specific HIV-1 3′ss A3 is critical for HIV-1 replication. Retrovirology.

[194] Erkelenz S., Theiss S., Otte M., Widera M., Peter J.O., Schaal H. (2014). Genomic HEXploring allows landscaping of novel potential splicing regulatory elements. Nucleic Acids Res..

[195] Zahler A.M., Damgaard C.K., Kjems J., Caputi M. (2004). SC35 and heterogeneous nuclear ribonucleoprotein A/B proteins bind to a juxtaposed exonic splicing enhancer/exonic splicing silencer element to regulate HIV-1 tat exon 2 splicing. J. Biol. Chem..

[196] Amendt B.A., Hesslein D., Chang L.J., Stoltzfus C.M. (1994). Presence of negative and positive cis-acting RNA splicing elements within and flanking the first tat coding exon of human immunodeficiency virus type 1. Mol. Cell. Biol..

[197] Caputi M., Mayeda A., Krainer A.R., Zahler A.M. (1999). hnRNP A/B proteins are required for inhibition of HIV-1 pre-mRNA splicing. EMBO J..

[198] Si Z., Amendt B.A., Stoltzfus C.M. (1997). Splicing efficiency of human immunodeficiency virus type 1 tat RNA is determined by both a suboptimal 3′ splice site and a 10 nucleotide exon splicing silencer element located within tat exon 2. Nucleic Acids Res..

[199] Jacquenet S., Mereau A., Bilodeau P.S., Damier L., Stoltzfus C.M., Branlant C. (2001). A second exon splicing silencer within human immunodeficiency virus type 1 tat exon 2 represses splicing of Tat mRNA and binds protein hnRNP H. J. Biol. Chem..

[200] Tange T.O., Damgaard C.K., Guth S., Valcarcel J., Kjems J. (2001). The hnRNP A1 protein regulates HIV-1 tat splicing via a novel intron silencer element. EMBO J..

[201] Huelga S.C., Vu A.Q., Arnold J.D., Liang T.Y., Liu P.P., Yan B.Y., Donohue J.P., Shiue L., Hoon S., Brenner S. (2012). Integrative genome-wide analysis reveals cooperative regulation of alternative splicing by hnRNP proteins. Cell Rep..

[202] Buratti E., Brindisi A., Giombi M., Tisminetzky S., Ayala Y.M., Baralle F.E. (2005). TDP-43 binds heterogeneous nuclear ribonucleoprotein A/B through its C-terminal tail: An important region for the inhibition of cystic fibrosis transmembrane conductance regulator exon 9 splicing. J. Biol. Chem..

[203] Hutchison S., LeBel C., Blanchette M., Chabot B. (2002). Distinct sets of adjacent heterogeneous nuclear ribonucleoprotein (hnRNP) A1/A2 binding sites control 5′ splice site selection in the hnRNP A1 mRNA precursor. J. Biol. Chem..

[204] Blanchette M., Green R.E., MacArthur S., Brooks A.N., Brenner S.E., Eisen M.B., Rio D.C. (2009). Genome-wide analysis of alternative pre-mRNA splicing and RNA-binding specificities of the Drosophila hnRNP A/B family members. Mol. Cell.

[205] Pasquereau S., Kumar A., Herbein G. (2017). Targeting TNF and TNF Receptor Pathway in HIV-1 Infection: From Immune Activation to Viral Reservoirs. Viruses.

[206] Dutilleul A., Rodari A., Van Lint C. (2020). Depicting HIV-1 Transcriptional Mechanisms: A Summary of What We Know. Viruses.

[207] Sonza S., Mutimer H.P., O‘Brien K., Ellery P., Howard J.L., Axelrod J.H., Deacon N.J., Crowe S.M., Purcell D.F. (2002). Selectively reduced tat mRNA heralds the decline in productive human immunodeficiency virus type 1 infection in monocyte-derived macrophages. J. Virol..

[208] Moron-Lopez S., Telwatte S., Sarabia I., Battivelli E., Montano M., Macedo A.B., Aran D., Butte A.J., Jones R.B., Bosque A. (2020). Human splice factors contribute to latent HIV infection in primary cell models and blood CD4+ T cells from ART-treated individuals. PLoS Pathog..

[209] Scalabrin M., Frasson I., Ruggiero E., Perrone R., Tosoni E., Lago S., Tassinari M., Palu G., Richter S.N. (2017). The cellular protein hnRNP A2/B1 enhances HIV-1 transcription by unfolding LTR promoter G-quadruplexes. Sci. Rep..

[210] Dahal S., Clayton K., Been T., Fernet-Brochu R., Ocando A.V., Balachandran A., Poirier M., Maldonado R.K., Shkreta L., Boligan K.F. (2022). Opposing roles of CLK SR kinases in controlling HIV-1 gene expression and latency. Retrovirology.

[211] Pasternak A.O., Berkhout B. (2021). The Splice of Life: Does RNA Processing Have a Role in HIV-1 Persistence?. Viruses.

[212] Erkelenz S., Mueller W.F., Evans M.S., Busch A., Schoneweis K., Hertel K.J., Schaal H. (2013). Position-dependent splicing activation and repression by SR and hnRNP proteins rely on common mechanisms. RNA.

[213] Fu X.D., Ares M. (2014). Context-dependent control of alternative splicing by RNA-binding proteins. Nat. Rev. Genet..

[214] Tomezsko P.J., Corbin V.D.A., Gupta P., Swaminathan H., Glasgow M., Persad S., Edwards M.D., McIntosh L., Papenfuss A.T., Emery A. (2020). Determination of RNA structural diversity and its role in HIV-1 RNA splicing. Nature.

[215] Barash Y., Calarco J.A., Gao W., Pan Q., Wang X., Shai O., Blencowe B.J., Frey B.J. (2010). Deciphering the splicing code. Nature.

[216] Wang Z., Burge C.B. (2008). Splicing regulation: From a parts list of regulatory elements to an integrated splicing code. RNA.

[217] Widera M., Erkelenz S., Hillebrand F., Krikoni A., Widera D., Kaisers W., Deenen R., Gombert M., Dellen R., Pfeiffer T. (2013). An Intronic G Run within HIV-1 Intron 2 Is Critical for Splicing Regulation of vif mRNA. J. Virol..

[218] Widera M., Hillebrand F., Erkelenz S., Vasudevan A., Munk C., Schaal H. (2014). A functional conserved intronic G run in HIV-1 intron 3 is critical to counteract APOBEC3G-mediated host restriction. Retrovirology.

[219] Exline C.M., Feng Z., Stoltzfus C.M. (2008). Negative and positive mRNA splicing elements act competitively to regulate human immunodeficiency virus type 1 vif gene expression. J. Virol..

[220] Wentz M.P., Moore B.E., Cloyd M.W., Berget S.M., Donehower L.A. (1997). A naturally arising mutation of a potential silencer of exon splicing in human immunodeficiency virus type 1 induces dominant aberrant splicing and arrests virus production. J. Virol..

[221] Madsen J.M., Stoltzfus C.M. (2005). An exonic splicing silencer downstream of the 3′ splice site A2 is required for efficient human immunodeficiency virus type 1 replication. J. Virol..

[222] Asang C., Erkelenz S., Schaal H. (2012). The HIV-1 major splice donor D1 is activated by splicing enhancer elements within the leader region and the p17-inhibitory sequence. Virology.

[223] Mikaelian I., Krieg M., Gait M.J., Karn J. (1996). Interactions of INS (CRS) elements and the splicing machinery regulate the production of Rev-responsive mRNAs. J. Mol. Biol..

[224] Schaub M.C., Lopez S.R., Caputi M. (2007). Members of the heterogeneous nuclear ribonucleoprotein H family activate splicing of an HIV-1 splicing substrate by promoting formation of ATP-dependent spliceosomal complexes. J. Biol. Chem..

[225] Brillen A.L., Walotka L., Hillebrand F., Muller L., Widera M., Theiss S., Schaal H. (2017). Analysis of competing HIV-1 splice donor sites uncovers a tight cluster of splicing regulatory elements within exon 2/2b. J. Virol..

[226] Bilodeau P.S., Domsic J.K., Mayeda A., Krainer A.R., Stoltzfus C.M. (2001). RNA splicing at human immunodeficiency virus type 1 3′ splice site A2 is regulated by binding of hnRNP A/B proteins to an exonic splicing silencer element. J. Virol..

[227] Domsic J.K., Wang Y., Mayeda A., Krainer A.R., Stoltzfus C.M. (2003). Human immunodeficiency virus type 1 hnRNP A/B-dependent exonic splicing silencer ESSV antagonizes binding of U2AF65 to viral polypyrimidine tracts. Mol. Cell. Biol..

[228] Hillebrand F., Peter J.O., Brillen A.L., Otte M., Schaal H., Erkelenz S. (2017). Differential hnRNP D isoform incorporation may confer plasticity to the ESSV-mediated repressive state across HIV-1 exon 3. Biochim. Biophys. Acta.

[229] Erkelenz S. (2013). Regulation of Human and HIV-1 Splice Sites. Ph.D. Thesis.

[230] Tsuruno C., Ohe K., Kuramitsu M., Kohma T., Takahama Y., Hamaguchi Y., Hamaguchi I., Okuma K. (2011). HMGA1a is involved in specific splice site regulation of human immunodeficiency virus type 1. Biochem. Biophys. Res. Commun..

[231] Tange T., Kjems J. (2001). SF2/ASF binds to a splicing enhancer in the third HIV-1 tat exon and stimulates U2AF binding independently of the RS domain. J. Mol. Biol..

[232] Amendt B.A., Si Z.H., Stoltzfus C.M. (1995). Presence of exon splicing silencers within human immunodeficiency virus type 1 tat exon 2 and tat-rev exon 3: Evidence for inhibition mediated by cellular factors. Mol. Cell. Biol..

[233] Si Z.H., Rauch D., Stoltzfus C.M. (1998). The exon splicing silencer in human immunodeficiency virus type 1 Tat exon 3 is bipartite and acts early in spliceosome assembly. Mol. Cell. Biol..

[234] Balachandran A., Ming L., Cochrane A., Parent L.J. (2018). Teetering on the Edge. Retrovirus-Cell Interactions.

[235] Jordan-Paiz A., Nevot M., Lamkiewicz K., Lataretu M., Franco S., Marz M., Martinez M.A. (2020). HIV-1 Lethality and Loss of Env Protein Expression Induced by Single Synonymous Substitutions in the Virus Genome Intronic-Splicing Silencer. J. Virol..

[236] Muller L., Moskorz W., Brillen A.L., Hillebrand F., Ostermann P.N., Kiel N., Walotka L., Ptok J., Timm J., Lubke N. (2021). Altered HIV-1 mRNA Splicing Due to Drug-Resistance-Associated Mutations in Exon 2/2b. Int. J. Mol. Sci..

[237] Doi N., Koma T., Adachi A., Nomaguchi M. (2019). Expression Level of HIV-1 Vif Can Be Fluctuated by Natural Nucleotide Variations in the vif-Coding and Regulatory SA1D2prox Sequences of the Proviral Genome. Front. Microbiol..

[238] Nguyen Quang N., Goudey S., Segeral E., Mohammad A., Lemoine S., Blugeon C., Versapuech M., Paillart J.C., Berlioz-Torrent C., Emiliani S. (2020). Dynamic nanopore long-read sequencing analysis of HIV-1 splicing events during the early steps of infection. Retrovirology.

[239] Koma T., Doi N., Le B.Q., Kondo T., Ishizue M., Tokaji C., Tsukada C., Adachi A., Nomaguchi M. (2023). Involvement of a Rarely Used Splicing SD2b Site in the Regulation of HIV-1 vif mRNA Production as Revealed by a Growth-Adaptive Mutation. Viruses.

[240] Engeland C.E., Brown N.P., Borner K., Schumann M., Krause E., Kaderali L., Muller G.A., Krausslich H.G. (2014). Proteome analysis of the HIV-1 Gag interactome. Virology.

[241] Grewe B., Vogt C., Horstkotter T., Tippler B., Xiao H., Muller B., Uberla K., Wagner R., Asbach B., Bohne J. (2021). The HIV 5′ Gag Region Displays a Specific Nucleotide Bias Regulating Viral Splicing and Infectivity. Viruses.

[242] Kim G.N., Yu K.L., Kim H.I., You J.C. (2022). Investigation of the effect of SRSF9 overexpression on HIV-1 production. BMB Rep..

[243] Asang C., Hauber I., Schaal H. (2008). Insights into the selective activation of alternatively used splice acceptors by the human immunodeficiency virus type-1 bidirectional splicing enhancer. Nucleic Acids Res..

[244] Robberson B.L., Cote G.J., Berget S.M. (1990). Exon definition may facilitate splice site selection in RNAs with multiple exons. Mol. Cell. Biol..

[245] Taniguchi I., Mabuchi N., Ohno M. (2014). HIV-1 Rev protein specifies the viral RNA export pathway by suppressing TAP/NXF1 recruitment. Nucleic Acids Res..

[246] Hautbergue G.M., Hung M.L., Golovanov A.P., Lian L.Y., Wilson S.A. (2008). Mutually exclusive interactions drive handover of mRNA from export adaptors to TAP. Proc. Natl. Acad. Sci. USA.

[247] Chen S., Wang R., Zheng D., Zhang H., Chang X., Wang K., Li W., Fan J., Tian B., Cheng H. (2019). The mRNA Export Receptor NXF1 Coordinates Transcriptional Dynamics, Alternative Polyadenylation, and mRNA Export. Mol. Cell.

[248] Pacheco-Fiallos B., Vorlander M.K., Riabov-Bassat D., Fin L., O‘Reilly F.J., Ayala F.I., Schellhaas U., Rappsilber J., Plaschka C. (2023). mRNA recognition and packaging by the human transcription-export complex. Nature.

[249] Puhringer T., Hohmann U., Fin L., Pacheco-Fiallos B., Schellhaas U., Brennecke J., Plaschka C. (2020). Structure of the human core transcription-export complex reveals a hub for multivalent interactions. Elife.

[250] Viphakone N., Hautbergue G.M., Walsh M., Chang C.T., Holland A., Folco E.G., Reed R., Wilson S.A. (2012). TREX exposes the RNA-binding domain of Nxf1 to enable mRNA export. Nat. Commun..

[251] Strasser K., Masuda S., Mason P., Pfannstiel J., Oppizzi M., Rodriguez-Navarro S., Rondon A.G., Aguilera A., Struhl K., Reed R. (2002). TREX is a conserved complex coupling transcription with messenger RNA export. Nature.

[252] Viphakone N., Sudbery I., Griffith L., Heath C.G., Sims D., Wilson S.A. (2019). Co-transcriptional Loading of RNA Export Factors Shapes the Human Transcriptome. Mol. Cell.

[253] Singh G., Kucukural A., Cenik C., Leszyk J.D., Shaffer S.A., Weng Z., Moore M.J. (2012). The cellular EJC interactome reveals higher-order mRNP structure and an EJC-SR protein nexus. Cell.

[254] Muller-McNicoll M., Botti V., de Jesus Domingues A.M., Brandl H., Schwich O.D., Steiner M.C., Curk T., Poser I., Zarnack K., Neugebauer K.M. (2016). SR proteins are NXF1 adaptors that link alternative RNA processing to mRNA export. Genes Dev..

[255] Zhong X.Y., Ding J.H., Adams J.A., Ghosh G., Fu X.D. (2009). Regulation of SR protein phosphorylation and alternative splicing by modulating kinetic interactions of SRPK1 with molecular chaperones. Genes Dev..

[256] Botti V., McNicoll F., Steiner M.C., Richter F.M., Solovyeva A., Wegener M., Schwich O.D., Poser I., Zarnack K., Wittig I. (2017). Cellular differentiation state modulates the mRNA export activity of SR proteins. J. Cell Biol..

[257] Huang Y., Yario T.A., Steitz J.A. (2004). A molecular link between SR protein dephosphorylation and mRNA export. Proc. Natl. Acad. Sci. USA.

[258] Twyffels L., Gueydan C., Kruys V. (2011). Shuttling SR proteins: More than splicing factors. FEBS J..

[259] Tintaru A.M., Hautbergue G.M., Hounslow A.M., Hung M.L., Lian L.Y., Craven C.J., Wilson S.A. (2007). Structural and functional analysis of RNA and TAP binding to SF2/ASF. EMBO Rep..

[260] Mahiet C., Swanson C.M. (2016). Control of HIV-1 gene expression by SR proteins. Biochem. Soc. Trans..

[261] Bejarano D.A., Peng K., Laketa V., Borner K., Jost K.L., Lucic B., Glass B., Lusic M., Muller B., Krausslich H.G. (2019). HIV-1 nuclear import in macrophages is regulated by CPSF6-capsid interactions at the nuclear pore complex. Elife.

[262] Jang S., Cook N.J., Pye V.E., Bedwell G.J., Dudek A.M., Singh P.K., Cherepanov P., Engelman A.N. (2019). Differential role for phosphorylation in alternative polyadenylation function versus nuclear import of SR-like protein CPSF6. Nucleic Acids Res..

[263] Xiao H., Wyler E., Milek M., Grewe B., Kirchner P., Ekici A., Silva A., Jungnickl D., Full F., Thomas M. (2021). CRNKL1 Is a Highly Selective Regulator of Intron-Retaining HIV-1 and Cellular mRNAs. mBio.

[264] Fischer U., Huber J., Boelens W.C., Mattaj I.W., Luhrmann R. (1995). The HIV-1 Rev activation domain is a nuclear export signal that accesses an export pathway used by specific cellular RNAs. Cell.

[265] Fernandes J., Jayaraman B., Frankel A. (2012). The HIV-1 Rev response element: An RNA scaffold that directs the cooperative assembly of a homo-oligomeric ribonucleoprotein complex. RNA Biol..

[266] Askjaer P., Jensen T.H., Nilsson J., Englmeier L., Kjems J. (1998). The specificity of the CRM1-Rev nuclear export signal interaction is mediated by RanGTP. J. Biol. Chem..

[267] Booth D.S., Cheng Y., Frankel A.D. (2014). The export receptor Crm1 forms a dimer to promote nuclear export of HIV RNA. Elife.

[268] Port S.A., Monecke T., Dickmanns A., Spillner C., Hofele R., Urlaub H., Ficner R., Kehlenbach R.H. (2015). Structural and Functional Characterization of CRM1-Nup214 Interactions Reveals Multiple FG-Binding Sites Involved in Nuclear Export. Cell Rep..

[269] Banerjee A., Benjamin R., Banerjee S. (2013). Impact of viral factors on subcellular distribution and RNA export activity of HIV-1 rev in astrocytes 1321N1. PLoS ONE.

[270] Brighty D.W., Rosenberg M. (1994). A cis-acting repressive sequence that overlaps the Rev-responsive element of human immunodeficiency virus type 1 regulates nuclear retention of env mRNAs independently of known splice signals. Proc. Natl. Acad. Sci. USA.

[271] Sodroski J., Goh W.C., Rosen C., Dayton A., Terwilliger E., Haseltine W. (1986). A second post-transcriptional trans-activator gene required for HTLV-III replication. Nature.

[272] Terwilliger E., Burghoff R., Sia R., Sodroski J., Haseltine W., Rosen C. (1988). The art gene product of human immunodeficiency virus is required for replication. J. Virol..

[273] Suh D., Seguin B., Atkinson S., Ozdamar B., Staffa A., Emili A., Mouland A., Cochrane A. (2003). Mapping of determinants required for the function of the HIV-1 env nuclear retention sequence. Virology.

[274] Pinol-Roma S. (1997). HnRNP proteins and the nuclear export of mRNA. Semin. Cell Dev. Biol..

[275] Nakielny S., Dreyfuss G. (1996). The hnRNP C proteins contain a nuclear retention sequence that can override nuclear export signals. J. Cell Biol..

[276] Gordon H., Ajamian L., Valiente-Echeverria F., Levesque K., Rigby W.F., Mouland A.J. (2013). Depletion of hnRNP A2/B1 overrides the nuclear retention of the HIV-1 genomic RNA. RNA Biol..

[277] Ryan V.H., Watters S., Amaya J., Khatiwada B., Venditti V., Naik M.T., Fawzi N.L. (2020). Weak binding to the A2RE RNA rigidifies hnRNPA2 RRMs and reduces liquid-liquid phase separation and aggregation. Nucleic Acids Res..

[278] Najera I., Krieg M., Karn J. (1999). Synergistic stimulation of HIV-1 rev-dependent export of unspliced mRNA to the cytoplasm by hnRNP A1. J. Mol. Biol..

[279] Izaurralde E., Jarmolowski A., Beisel C., Mattaj I.W., Dreyfuss G., Fischer U. (1997). A role for the M9 transport signal of hnRNP A1 in mRNA nuclear export. J. Cell Biol..

[280] Siomi M.C., Eder P.S., Kataoka N., Wan L., Liu Q., Dreyfuss G. (1997). Transportin-mediated nuclear import of heterogeneous nuclear RNP proteins. J. Cell Biol..

[281] Black A.C., Luo J., Chun S., Bakker A., Fraser J.K., Rosenblatt J.D. (1996). Specific binding of polypyrimidine tract binding protein and hnRNP A1 to HIV-1 CRS elements. Virus Genes.

[282] Zolotukhin A.S., Michalowski D., Bear J., Smulevitch S.V., Traish A.M., Peng R., Patton J., Shatsky I.N., Felber B.K. (2003). PSF acts through the human immunodeficiency virus type 1 mRNA instability elements to regulate virus expression. Mol. Cell. Biol..

[283] Yandrapally S., Sarkar S., Banerjee S. (2023). HIV-1 Tat commandeers nuclear export of Rev-viral RNA complex by controlling hnRNPA2-mediated splicing. J. Virol..

[284] Cazalla D., Zhu J., Manche L., Huber E., Krainer A.R., Caceres J.F. (2002). Nuclear export and retention signals in the RS domain of SR proteins. Mol. Cell. Biol..

[285] Jacks T., Varmus H.E. (1985). Expression of the Rous sarcoma virus pol gene by ribosomal frameshifting. Science.

[286] Namy O., Moran S.J., Stuart D.I., Gilbert R.J., Brierley I. (2006). A mechanical explanation of RNA pseudoknot function in programmed ribosomal frameshifting. Nature.

[287] Jacks T., Power M.D., Masiarz F.R., Luciw P.A., Barr P.J., Varmus H.E. (1988). Characterization of ribosomal frameshifting in HIV-1 gag-pol expression. Nature.

[288] Nam S.H., Kidokoro M., Shida H., Hatanaka M. (1988). Processing of gag precursor polyprotein of human T-cell leukemia virus type I by virus-encoded protease. J. Virol..

[289] Marra M.A., Jones S.J., Astell C.R., Holt R.A., Brooks-Wilson A., Butterfield Y.S., Khattra J., Asano J.K., Barber S.A., Chan S.Y. (2003). The Genome sequence of the SARS-associated coronavirus. Science.

[290] Bhatt P.R., Scaiola A., Loughran G., Leibundgut M., Kratzel A., Meurs R., Dreos R., O‘Connor K.M., McMillan A., Bode J.W. (2021). Structural basis of ribosomal frameshifting during translation of the SARS-CoV-2 RNA genome. Science.

[291] Belew A.T., Meskauskas A., Musalgaonkar S., Advani V.M., Sulima S.O., Kasprzak W.K., Shapiro B.A., Dinman J.D. (2014). Ribosomal frameshifting in the CCR5 mRNA is regulated by miRNAs and the NMD pathway. Nature.

[292] Kang H. (1998). Direct structural evidence for formation of a stem-loop structure involved in ribosomal frameshifting in human immunodeficiency virus type 1. Biochim. Biophys. Acta.

[293] Mikl M., Pilpel Y., Segal E. (2020). High-throughput interrogation of programmed ribosomal frameshifting in human cells. Nat. Commun..

[294] Cen S., Niu M., Saadatmand J., Guo F., Huang Y., Nabel G.J., Kleiman L. (2004). Incorporation of pol into human immunodeficiency virus type 1 Gag virus-like particles occurs independently of the upstream Gag domain in Gag-pol. J. Virol..

[295] Shehu-Xhilaga M., Crowe S.M., Mak J. (2001). Maintenance of the Gag/Gag-Pol ratio is important for human immunodeficiency virus type 1 RNA dimerization and viral infectivity. J. Virol..

[296] Napthine S., Hill C.H., Nugent H.C.M., Brierley I. (2021). Modulation of Viral Programmed Ribosomal Frameshifting and Stop Codon Readthrough by the Host Restriction Factor Shiftless. Viruses.

[297] Zimmer M.M., Kibe A., Rand U., Pekarek L., Ye L., Buck S., Smyth R.P., Cicin-Sain L., Caliskan N. (2021). The short isoform of the host antiviral protein ZAP acts as an inhibitor of SARS-CoV-2 programmed ribosomal frameshifting. Nat. Commun..

[298] Wang X., Xuan Y., Han Y., Ding X., Ye K., Yang F., Gao P., Goff S.P., Gao G. (2019). Regulation of HIV-1 Gag-Pol Expression by Shiftless, an Inhibitor of Programmed -1 Ribosomal Frameshifting. Cell.

[299] Jager N., Ayyub S.A., Korniy N., Peske F., Hoffmann M., Rodnina M.V., Pohlmann S. (2022). Mutagenic Analysis of the HIV Restriction Factor Shiftless. Viruses.

[300] Yu D., Zhao Y., Pan J., Yang X., Liang Z., Xie S., Cao R. (2021). C19orf66 Inhibits Japanese Encephalitis Virus Replication by Targeting -1 PRF and the NS3 Protein. Virol. Sin..

[301] Suzuki Y., Chin W.X., Han Q., Ichiyama K., Lee C.H., Eyo Z.W., Ebina H., Takahashi H., Takahashi C., Tan B.H. (2016). Characterization of RyDEN (C19orf66) as an Interferon-Stimulated Cellular Inhibitor against Dengue Virus Replication. PLoS Pathog..

[302] Wu Y., Yang X., Yao Z., Dong X., Zhang D., Hu Y., Zhang S., Lin J., Chen J., An S. (2020). C19orf66 interrupts Zika virus replication by inducing lysosomal degradation of viral NS3. PLoS Neglected Trop. Dis..

[303] Kinast V., Plociennikowska A., Anggakusuma, Bracht T., Todt D., Brown R.J.P., Boldanova T., Zhang Y., Bruggemann Y., Friesland M. (2020). C19orf66 is an interferon-induced inhibitor of HCV replication that restricts formation of the viral replication organelle. J. Hepatol..

[304] Rodriguez W., Mehrmann T., Hatfield D., Muller M. (2022). Shiftless Restricts Viral Gene Expression and Influences RNA Granule Formation during Kaposi’s Sarcoma-Associated Herpesvirus Lytic Replication. J. Virol..

[305] Gao G., Guo X., Goff S.P. (2002). Inhibition of retroviral RNA production by ZAP, a CCCH-type zinc finger protein. Science.

[306] Müller S., Möller P., Bick M.J., Wurr S., Becker S., Günther S., Kümmerer B.M. (2007). Inhibition of filovirus replication by the zinc finger antiviral protein. J. Virol..

[307] Tang Q., Wang X., Gao G. (2017). The Short Form of the Zinc Finger Antiviral Protein Inhibits Influenza A Virus Protein Expression and Is Antagonized by the Virus-Encoded NS1. J. Virol..

[308] Chiu H.P., Chiu H., Yang C.F., Lee Y.L., Chiu F.L., Kuo H.C., Lin R.J., Lin Y.L. (2018). Inhibition of Japanese encephalitis virus infection by the host zinc-finger antiviral protein. PLoS Pathog..

[309] Zhu Y., Chen G., Lv F., Wang X., Ji X., Xu Y., Sun J., Wu L., Zheng Y.T., Gao G. (2011). Zinc-finger antiviral protein inhibits HIV-1 infection by selectively targeting multiply spliced viral mRNAs for degradation. Proc. Natl. Acad. Sci. USA.

[310] Trono D., Van Lint C., Rouzioux C., Verdin E., Barre-Sinoussi F., Chun T.W., Chomont N. (2010). HIV persistence and the prospect of long-term drug-free remissions for HIV-infected individuals. Science.

[311] Chun T.W., Moir S., Fauci A.S. (2015). HIV reservoirs as obstacles and opportunities for an HIV cure. Nat. Immunol..

[312] Pennings P.S. (2013). HIV Drug Resistance: Problems and Perspectives. Infect. Dis. Rep..

[313] Saayman S.M., Lazar D.C., Scott T.A., Hart J.R., Takahashi M., Burnett J.C., Planelles V., Morris K.V., Weinberg M.S. (2016). Potent and Targeted Activation of Latent HIV-1 Using the CRISPR/dCas9 Activator Complex. Mol. Ther. J. Am. Soc. Gene Ther..

[314] Soret J., Bakkour N., Maire S., Durand S., Zekri L., Gabut M., Fic W., Divita G., Rivalle C., Dauzonne D. (2005). Selective modification of alternative splicing by indole derivatives that target serine-arginine-rich protein splicing factors. Proc. Natl. Acad. Sci. USA.

[315] Bakkour N., Lin Y.L., Maire S., Ayadi L., Mahuteau-Betzer F., Nguyen C.H., Mettling C., Portales P., Grierson D., Chabot B. (2007). Small-molecule inhibition of HIV pre-mRNA splicing as a novel antiretroviral therapy to overcome drug resistance. PLoS Pathog..

[316] Soret J., Gabut M., Tazi J. (2006). SR proteins as potential targets for therapy. Prog. Mol. Subcell. Biol..

[317] Cheung P.K., Horhant D., Bandy L.E., Zamiri M., Rabea S.M., Karagiosov S.K., Matloobi M., McArthur S., Harrigan P.R., Chabot B. (2016). A Parallel Synthesis Approach to the Identification of Novel Diheteroarylamide-Based Compounds Blocking HIV Replication: Potential Inhibitors of HIV-1 Pre-mRNA Alternative Splicing. J. Med. Chem..

[318] Shkreta L., Blanchette M., Toutant J., Wilhelm E., Bell B., Story B.A., Balachandran A., Cochrane A., Cheung P.K., Harrigan P.R. (2017). Modulation of the splicing regulatory function of SRSF10 by a novel compound that impairs HIV-1 replication. Nucleic Acids Res..

[319] Tazi J., Bakkour N., Marchand V., Ayadi L., Aboufirassi A., Branlant C. (2010). Alternative splicing: Regulation of HIV-1 multiplication as a target for therapeutic action. FEBS J..

[320] Wong R.W., Balachandran A., Ostrowski M.A., Cochrane A. (2013). Digoxin suppresses HIV-1 replication by altering viral RNA processing. PLoS Pathog..

[321] Wong R.W., Balachandran A., Haaland M., Stoilov P., Cochrane A. (2013). Characterization of novel inhibitors of HIV-1 replication that function via alteration of viral RNA processing and rev function. Nucleic Acids Res..

[322] Campos N., Myburgh R., Garcel A., Vautrin A., Lapasset L., Nadal E.S., Mahuteau-Betzer F., Najman R., Fornarelli P., Tantale K. (2015). Long lasting control of viral rebound with a new drug ABX464 targeting Rev—Mediated viral RNA biogenesis. Retrovirology.

[323] Hammond S.M., Caudy A.A., Hannon G.J. (2001). Post-transcriptional gene silencing by double-stranded RNA. Nat. Rev. Genet..

[324] Meister G., Tuschl T. (2004). Mechanisms of gene silencing by double-stranded RNA. Nature.

[325] Asparuhova M.B., Marti G., Liu S., Serhan F., Trono D., Schumperli D. (2007). Inhibition of HIV-1 multiplication by a modified U7 snRNA inducing Tat and Rev exon skipping. J. Gene Med..

[326] Asparuhova M.B., Barde I., Trono D., Schranz K., Schumperli D. (2008). Development and characterization of a triple combination gene therapy vector inhibiting HIV-1 multiplication. J. Gene Med..

[327] Chen M.J., Gatignol A., Scarborough R.J. (2023). The discovery and development of RNA-based therapies for treatment of HIV-1 infection. Expert Opin. Drug Discov..

[328] Mandal D., Feng Z., Stoltzfus C.M. (2010). Excessive RNA splicing and inhibition of HIV-1 replication induced by modified U1 small nuclear RNAs. J. Virol..

[329] Del Corpo O., Goguen R.P., Malard C.M.G., Daher A., Colby-Germinario S., Scarborough R.J., Gatignol A. (2019). A U1i RNA that Enhances HIV-1 RNA Splicing with an Elongated Recognition Domain Is an Optimal Candidate for Combination HIV-1 Gene Therapy. Mol. Ther. Nucleic Acids.

[330] Grunweller A., Hartmann R.K. (2007). Locked nucleic acid oligonucleotides: The next generation of antisense agents?. BioDrugs Clin. Immunother. Biopharm. Gene Ther..

[331] Lundin K.E., Hojland T., Hansen B.R., Persson R., Bramsen J.B., Kjems J., Koch T., Wengel J., Smith C.I. (2013). Biological activity and biotechnological aspects of locked nucleic acids. Adv. Genet..

[332] Stein C.A., Hansen J.B., Lai J., Wu S., Voskresenskiy A., Hog A., Worm J., Hedtjarn M., Souleimanian N., Miller P. (2010). Efficient gene silencing by delivery of locked nucleic acid antisense oligonucleotides, unassisted by transfection reagents. Nucleic Acids Res..

[333] Zhang Y., Qu Z., Kim S., Shi V., Liao B., Kraft P., Bandaru R., Wu Y., Greenberger L.M., Horak I.D. (2011). Down-modulation of cancer targets using locked nucleic acid (LNA)-based antisense oligonucleotides without transfection. Gene Ther..

[334] Hillebrand F., Ostermann P.N., Muller L., Degrandi D., Erkelenz S., Widera M., Pfeffer K., Schaal H. (2019). Gymnotic Delivery of LNA Mixmers Targeting Viral SREs Induces HIV-1 mRNA Degradation. Int. J. Mol. Sci..

[335] Arzumanov A., Walsh A.P., Rajwanshi V.K., Kumar R., Wengel J., Gait M.J. (2001). Inhibition of HIV-1 Tat-dependent trans activation by steric block chimeric 2′-O-methyl/LNA oligoribonucleotides. Biochemistry.

[336] Lebars I., Richard T., Di Primo C., Toulme J.J. (2007). LNA derivatives of a kissing aptamer targeted to the trans-activating responsive RNA element of HIV-1. Blood Cells Mol. Dis..

[337] Jakobsen M.R., Haasnoot J., Wengel J., Berkhout B., Kjems J. (2007). Efficient inhibition of HIV-1 expression by LNA modified antisense oligonucleotides and DNAzymes targeted to functionally selected binding sites. Retrovirology.

[338] Holmes S.C., Arzumanov A.A., Gait M.J. (2003). Steric inhibition of human immunodeficiency virus type-1 Tat-dependent trans-activation in vitro and in cells by oligonucleotides containing 2′-O-methyl G-clamp ribonucleoside analogues. Nucleic Acids Res..

[339] Uleri E., Beltrami S., Gordon J., Dolei A., Sariyer I.K. (2011). Extinction of Tumor Antigen Expression by SF2/ASF in JCV-Transformed Cells. Genes Cancer.

[340] Uleri E., Regan P., Dolei A., Sariyer I.K. (2013). SF2/ASF binding region within JC virus NCCR limits early gene transcription in glial cells. Virol. J..

[341] Sariyer I.K., Khalili K. (2011). Regulation of human neurotropic JC virus replication by alternative splicing factor SF2/ASF in glial cells. PLoS ONE.

[342] Sariyer I.K., Sariyer R., Otte J., Gordon J. (2016). Pur-Alpha Induces JCV Gene Expression and Viral Replication by Suppressing SRSF1 in Glial Cells. PLoS ONE.

[343] McPhillips M.G., Veerapraditsin T., Cumming S.A., Karali D., Milligan S.G., Boner W., Morgan I.M., Graham S.V. (2004). SF2/ASF binds the human papillomavirus type 16 late RNA control element and is regulated during differentiation of virus-infected epithelial cells. J. Virol..

[344] Molin M., Akusjarvi G. (2000). Overexpression of essential splicing factor ASF/SF2 blocks the temporal shift in adenovirus pre-mRNA splicing and reduces virus progeny formation. J. Virol..

[345] Ofori L.O., Hilimire T.A., Bennett R.P., Brown N.W., Smith H.C., Miller B.L. (2014). High-affinity recognition of HIV-1 frameshift-stimulating RNA alters frameshifting in vitro and interferes with HIV-1 infectivity. J. Med. Chem..

[346] Anokhina V.S., Miller B.L. (2021). Targeting Ribosomal Frameshifting as an Antiviral Strategy: From HIV-1 to SARS-CoV-2. Acc. Chem. Res..

[347] Knizhnik E., Chumakov S., Svetlova J., Pavlova I., Khodarovich Y., Brylev V., Severov V., Alieva R., Kozlovskaya L., Andreev D. (2023). Unwinding the SARS-CoV-2 Ribosomal Frameshifting Pseudoknot with LNA and G-Clamp-Modified Phosphorothioate Oligonucleotides Inhibits Viral Replication. Biomolecules.

